# Artificial Zymogen Based on Protein–Polymer Hybrids

**DOI:** 10.1021/acs.biomac.4c01079

**Published:** 2024-10-18

**Authors:** Hironobu Murata, Kriti Kapil, Bibifatima Kaupbayeva, Alan J. Russell, Jonathan S. Dordick, Krzysztof Matyjaszewski

**Affiliations:** † Department of Chemistry, Carnegie Mellon University, 4400 Fifth Avenue, Pittsburgh, Pennsylvania 15213, United States; ‡ National Laboratory Astana, Nazarbayev University, Astana 010000, Kazakhstan; § Amgen Research, 1 Amgen Center Drive, Thousand Oaks, California 91320, United States; ∥ Department of Chemical and Biological Engineering, Center for Biotechnology & Interdisciplinary Studies, Rensselaer Polytechnic Institute, Troy, New York 12180, United States

## Abstract

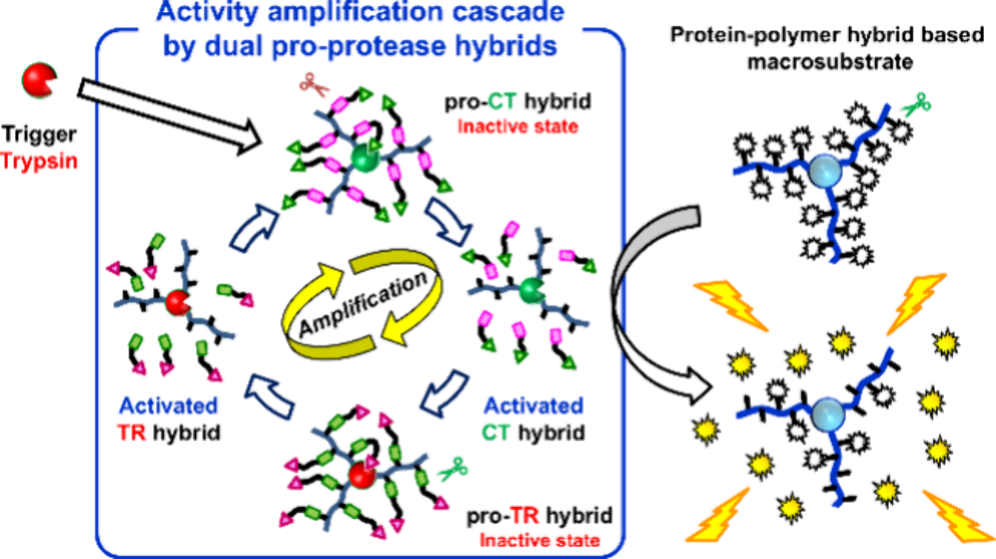

This study explores the synthesis and application of artificial zymogens using protein–polymer hybrids to mimic the controlled enzyme activation observed in natural zymogens. Pro-trypsin (pro-TR) and pro-chymotrypsin (pro-CT) hybrids were engineered by modifying the surfaces of trypsin (TR) and chymotrypsin (CT) with cleavable peptide inhibitors utilizing surface-initiated atom transfer radical polymerization. These hybrids exhibited 70 and 90% reductions in catalytic efficiency for pro-TR and pro-CT, respectively, due to the inhibitory effect of the grafted peptide inhibitors. The activation of pro-TR by CT and pro-CT by TR resulted in 1.5- and 2.5-fold increases in enzymatic activity, respectively. Furthermore, the activated hybrids triggered an enzyme activation cascade, enabling amplification of activity through a dual pro-protease hybrid system. This study highlights the potential of artificial zymogens for therapeutic interventions and biodetection platforms by harnessing enzyme activation cascades for precise control of catalytic activity.

## Introduction

Nature has developed sophisticated mechanisms by expressing enzymes as inactive proenzymes (zymogens), allowing for the controlled expression of enzyme function on demand and protecting itself from environmental damage caused by unnecessary enzyme activity.^1,2^ Zymogen activation mechanisms encompass diverse biochemical processes, including enzymatic or nonenzymatic cofactor-mediated transformations, autocatalytic conversions, and alterations induced by variations in pH. The use of proprotein formats extends across diverse biological pathways such as blood coagulation, apoptosis signaling networks, secretion of hormones and growth factors, digestion, and extracellular matrix remodeling.^3−8^ A notable instance occurs in proteolytic cascades, where enzymes like trypsin (TR) and chymotrypsin (CT) are synthesized as inactive precursor forms (trypsinogen and chymotrypsinogen, respectively) in the pancreas. Upon reaching the small intestine, trypsinogen is activated by enterokinase (also known as enteropeptidase, EP) through specific proteolytic cleavage to form activated TR. The TR then activates other TR, CT, and carboxypeptidases, which take part in the digestive process. However, trypsinogen is not activated by CT in nature.^9,10^ Inspired by nature, synthetic or artificial proenzyme cascade systems that precisely advance or amplify enzyme activation represent a new approach in biomedical research focused on targeting and controlling activity expression.

Covalent modifications of proteins with precisely engineered synthetic polymers represent a versatile strategy for modulating protein characteristics, including stability, activity, circulation longevity in biological fluids, as well as mitigating immunogenic response.^11−20^ Hence, these protein–polymer hybrids (PPH) find widespread utility across diverse domains such as medicine, biotechnology, and nanotechnology.^21−34^ Reversible deactivation radical polymerization (RDRP) techniques, notably atom transfer radical polymerization (ATRP) and reversible addition–fragmentation chain transfer (RAFT) offer attractive routes for generating well-defined protein and nucleic-acid biohybrids.^35−38^ Leveraging these methodologies affords precise control over polymerization kinetics, enabling tailored manipulation of polymer chain length and dispersity under benign conditions.^39−49^ The compatibility with a variety of monomers enhances the versatility and utility of these techniques in PPH synthesis, and new functionalities derived from modified polymers are added to PPH.^50−55^ The “grafting from” approach is a prominent method for synthesizing PPH. In this approach, small initiator molecules or chain transfer agents are covalently anchored onto the protein surface. Subsequently, polymer chains are directly grafted from these initiating sites.^56−69^ This methodology offers distinct advantages, facilitating controlled polymer growth while minimizing steric hindrance, thereby ensuring efficient conjugation.^70^ These advancements in RDRP have enabled high-throughput and solid-phase synthesis of PPH.^71−76^

Manipulation of protein surfaces with modified polymers can not only improve the pH and temperature stability of the protein but also tune the substrate affinity or inhibitory protein shielding effect through “molecular sieving” behavior.^77−83^ The “molecular sieving” behavior of modified polymers mainly depends on the grafting density and can be tuned by strategies such as multiheaded ATRP initiators,^84−86^ comb-shaped,^87^ or hyperbranched functional polymers.^77,88−90^ This approach has garnered significant interest in crafting “smart conjugates” by incorporating stimuli-responsive polymers capable of dynamic responses to environmental cues like temperature and pH.^30,55,91−97^ Furthermore, the molecular sieving effect engendered by the augmented excluded volume of hydrophilic synthetic polymers contributes substantially to the stability of hybrid proteins while mitigating protein–protein interactions.^79,82,98,99^ For instance, grafting comb-shaped polymers onto the surface of CT effectively prevents enzyme inhibition by protein inhibitors without compromising the enzyme’s catalytic activity in peptide substrate hydrolysis. Moreover, in comb-shaped polymer–protein hybrids featuring side chains susceptible to cleavage by digestive enzymes, post-treatment with digestive enzymes augments protein–protein interactions, thereby enabling controlled modulation of these interactions triggered by digestive enzymes.^87^ This augmentation of protein–protein interactions in PPH activated by digestive enzymes holds promise for applications, such as the preparation and activation of PPH-based artificial zymogens.

**Figure 1:**
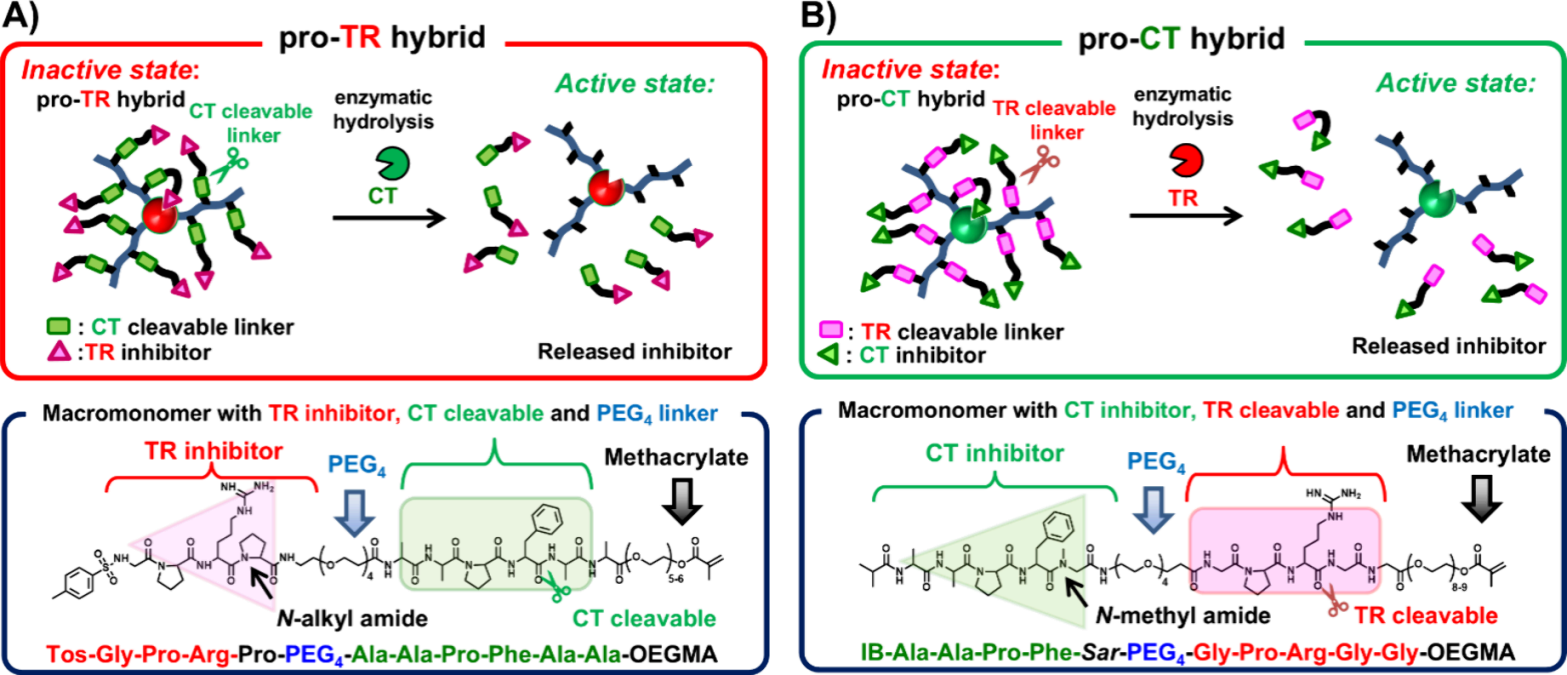
Concept of pro-protease hybrids and details of macromonomers. (A) Pro-TR hybrid synthesized using a macromonomer containing TR inhibitor and CT cleavable sites. (B) Pro-CT hybrid synthesized using a macromonomer containing CT inhibitor and TR cleavable sites.

In this study, we aimed to create an artificial zymogen with controllable activation by employing the technology of protein shielding using modified polymer chains and incorporating cleavable inhibitors into the polymer structure (Figure 1). TR and CT, well-known digestive enzymes, were selected as model enzymes. For example, TR specifically hydrolyzes the amide bond at the carboxyl side of basic amino acid residues such as arginine and lysine, while CT acts on amide bonds adjacent to aromatic amino acid residues like phenylalanine, tyrosine, and tryptophan. However, TR and CT lose their hydrolytic activity when the amide bond adjacent to a basic or aromatic amino acid lacks a proton, as seen with proline. These artificial pro-proteases exhibit minimal enzymatic activity due to the shielding effect provided by grafted polymer chains and the inhibitory action of bound peptide inhibitors. The enzymatic activity of pro-protease hybrids increases upon cleavage of the peptide inhibitor from the polymer chain by an exogenous trigger enzyme. Specifically, we engineered the artificial pro-TR hybrid to be activated by CT, diverging from the typical activation pathway of natural trypsinogen by active TR (Figure 1A)**.** Conversely, the artificial pro-CT hybrid was designed to mimic the activation mechanism observed in native chymotrypsinogen, activated by TR (Figure 1B)**.** Furthermore, considering the potential for an activated pro-protease hybrid to catalyze the activation of its counterpart pro-protease hybrid, this study delves into investigating activity amplification in dual pro-protease hybrids triggered by an active protease. This exploration provides insights into potential mechanisms for enhancing enzyme activation through engineered pro-protease hybrids, thus advancing applications in enzyme cascades and biocatalysis.

## Materials and Methods

α-Chymotrypsin from bovine pancreas, Type II (CT), trypsin from bovine pancreas, Type I (TR), and bovine serum albumin (BSA) were purchased from Sigma-Aldrich (St. Louis, MO). Copper chloride dihydrate (CuCl_2_·2H_2_O), 1,1,4,7,10,10-hexamethyl-triethylenetetramine (HMTETA), sodium ascorbate, poly(ethylene glycol) methacrylate (average *M*_n_ 360 and 500), isobutyryl chloride, *p*-toluenesulfonic acid monohydrate, succinic anhydride, triethylamine, palladium on carbon powder (10 wt %, Pd/C), ammonium formate, *N*-(3-(dimethylamino)propyl)-*N*′-ethylcarbodiimide hydrochloride (EDC·HCl), *N*-hydroxysuccinimide (NHS), 4-(dimethylamino)pyridine (DMAP), trifluoroacetic acid (TFA), hydrogen chloride solution (4 M in 1,4-dioxane), fluorescamine, bicinchoninic acid solution, and copper sulfate solution were purchased from Sigma-Aldrich. 3-[[2-(Methacryloyloxy)ethyl]-dimethylammonio]propionate (CBMA), l-proline benzyl ester hydrochloride (HCl·H-Pro-OBzl), l-Alanine methyl ester hydrochloride (HCl·H-Ala-OMe), glycine benzyl ester *p*-toluenesulfonate (TosOH·H-Gly-OBzl), and 4-(4,6-Dimethoxy-1,3,5-triazin-2-yl)-4-methylmorpholinium chloride (DMTMM) was purchased from TCI America (Philadelphia, PA)**.***N*-(*tert*-Butoxycarbonyl)-l-alanine (Boc-Ala-OH), *N*-(*tert*-butoxycarbonyl)-l-phenylalanine (Boc-Phe-OH), *N*-(*tert*-butoxycarbonyl)-glycine (Boc-Gly-OH), *N*_α_-(*tert*-butoxycarbonyl)-*N*_ω_-nitro-l-arginine (Boc-Arg(NO_2_)–OH), l-Phenylalanine *p*-nitroanilide (Phe-pNA), l-arginine-*p*-nitroanilide hydrochloride (Arg-pNA), and *N*-succinyl-l-Ala-l-Ala-l-Pro-l-Phe-*p*-nitroanilide (sucAAPFpNA) were purchased from Bachem (Torrance, CA). Sarcosine benzyl ester *p*-toluenesulfonate salt (TosOH**·**H-SarOBzl) was purchased from AmBeed (Arlington Heights, IL) *N*-Tosyl-glycyl-l-prolyl-l-arginine *p*-nitroanilide acetate salt (Tosyl-GPRpNA) was purchased from Cayman Chemical (Ann Arbor, MI). Amino-PEG_4_-*t*-butyl ester was purchased from BroadPharm (San Diego, CA). *N*-2-Bromo-2-methyl propionyl-β-alanine *N*-oxysuccinimide ester (NHS-ATRP Initiator) was prepared as described previously.^93^

### Synthesis of Macromonomers Containing the Protease Inhibitor and Cleavable Peptide Moieties

Macromonomers containing TR or CT inhibitors and cleavable peptide moieties were synthesized according to the previously reported method for the synthesis of macromonomers containing oligopeptides using a solution phase method (Supporting Information, Schemes S1 and S2).^87,100,101^

### Modification of the ATRP Initiator on the Surface of TR and CT

TR and CT macro ATRP initiator hybrids were synthesized using NHS-ATRP initiator and the number of ATRP initiators on CT or TR surface was determined by the fluorescamine assay according to previously reported methods.^87,93^

### Synthesis of the Pro-TR Hybrid Containing the TR Inhibitor and CT Cleavable Peptide Moieties

Pro-TR hybrid was synthesized using TR-ATRP macroinitiator (4.7 mg, 2.5 μmol of initiator groups), CBMA (52 mg, 225 μmol for targeted DP of 90), and Tos-Gly-Pro-Arg-Pro-PEG_4_-Ala-Ala-Pro-Phe-Ala-Ala-OEGMA solution in DMSO (46 mg, 25 μmol in 460 μL of DMSO for target DP 10) in phosphate-buffered saline (PBS) (4 mL) and DMSO (500 μL). The flask was sealed with a rubber septum, placed in an ice bath, and bubbled with argon for 30 min. In a separate vial, 25 mM CuCl_2_ solution (1.2 mL, 30 μmol of CuCl_2_) was bubbled under argon for 2 min. Sodium ascorbate (30 μL of 20 mg mL^–1^, 3 μmol) and HMTETA (9.7 μL, 36 μmol) were added to the deoxygenated CuCl_2_ solution and the mixture was bubbled for another 1 min. Deoxygenated copper catalyst solution (1 mL) was added to the solution of deoxygenated TR-macro initiator/CBMA/Tos-Gly-Pro-Arg-Pro-PEG_4_-Ala-Ala-Pro-Phe-Ala-Ala-OEGMA and allowed to react in a refrigerator for 1 h. The reaction was stopped upon exposure to air, and the pro-TR hybrid was purified through dialysis (50 kDa MWCO) against 25 mM sodium phosphate (pH 7.0) and deionized water in the refrigerator for 24 h and then lyophilized.

### Synthesis of the Pro-CT Hybrid Containing the CT Inhibitor and TR Cleavable Peptide Moieties

Pro-CT hybrid was synthesized using CT-ATRP macroinitiator (5.8 mg, 2.5 μmol of initiator groups), CBMA (52 mg, 225 μmol for targeted DP of 90), and IB-Ala-Ala-Pro-Phe-Sar-PEG_4_-Gly-Pro-Arg-Gly-Gly-OEGMA solution in DMSO (43 mg, 25 μmol in 430 μL of DMSO for target DP 10) in phosphate-buffered saline (PBS) (4 mL) and DMSO (500 μL). The subsequent steps were performed according to the procedure mentioned above.

### Synthesis of BSA-Polymer Hybrid-Based Macrosubstrates

The BSA polymer hybrid-based macrosubstrate, BSA-p(CBMA-*co*-MAOEG-AAPFpNA), containing the CT-sensitive peptide substrate with a pNA indicator in the side chain of the grafted polymer on the BSA surface was synthesized following a previously reported procedure.^87^

### ^1^H NMR Analysis of CT and TR–Polymer Hybrids

^1^H NMR spectra were recorded on a spectrometer (Bruker Avance III 500 MHz NMR Instrument) in the NMR facility located in the Center for Molecular Analysis, Carnegie Mellon University, Pittsburgh, PA, with deuterium oxide (D_2_O), DMSO-*d*_6_ and C*D*Cl_3_. Ten mg samples of the protein–polymer hybrid were dissolved in 500 μL of D_2_O.

### BCA Assay to Determine TR or CT Concentration in the Hybrid

The purified PPH (1.0 mg) was dissolved in deionized water, and the sample (25 μL) was mixed with a bicinchoninic acid (BCA) solution (1.0) and copper(II) sulfate solution (50:1 v:v). The solution was incubated at 60 °C for 15 min. The absorbance of the sample was recorded at 562 nm by using a UV–vis spectrometer. The weight % of TR or CT in the hybrid was determined by a comparison of the absorbance to a standard curve (native TR and CT).

### SEC-MALLS Characterizations of Hybrids

Dulbecco’s phosphate buffered saline without calcium chloride and magnesium chloride (pH 7.4) was used as solvent and eluent for all SEC-MALLS measurements using Agilent SEC system equipped with Waters Ultrahydrogel Linear column and coupled with MALLS, UV, and RI detectors (Wyatt Technology, USA). The sample concentration was about 2 mg mL^–1,^ and the injection load was 100 μL. The Astra software, version 8.0, was used to collect and process detector data (Wyatt Technology, USA). The refractive index increment d*n*/d*c* was determined by manual injection into the RI detector of samples with varied concentrations.

### Biocatalytic Assay for Pro-TR and Activated TR Hybrids

*N*-Tosyl-l-Gly-l-Pro-l-Arg-*p*-nitroanilide was used as a substrate for enzyme bioactivity assays. In a cuvette, 0.1 M Tris HCl and 20 mM calcium chloride buffer (890–990 μL, pH 8.0), substrate (0–100 μL, 2 mg/mL in DMSO), and enzyme (10 μL, 0.02 mg TR/mL in 100 mM Tris–HCl and 20 mM calcium chloride (pH 8.0), 8.4 nM as final concentration) were mixed for enzyme bioactivity assays. The rate of hydrolysis was determined by recording the increase in absorbance at 412 nm for the first 45 s after mixing. *K*_M_ and *k*_cat_ values were calculated using Enzfitter software when plotting substrate concentration versus initial hydrolysis velocity.

### Biocatalytic Assay for Pro-CT and Activated CT Hybrids

*N*-Succinyl-l-Ala-l-Ala-l-Pro-l-Phe-*p*-nitroanilide was used as a substrate for enzyme bioactivity assays. In a cuvette, 0.1 M sodium phosphate buffer (910–990 μL, pH 8.0), substrate (0–80 μL, 6 mg/mL in DMSO), and enzyme (10 μL, 0.1 mg CT/mL in 0.1 M sodium phosphate buffer (pH 8.0), 40 nM as final CT concentration) were mixed. The rate of hydrolysis was determined by recording the increase in absorbance at 412 nm for the first 45 s after mixing. *K*_M_ and *k*_cat_ values were calculated using the Enzfitter software when plotting substrate concentration versus initial hydrolysis velocity.

### Activation of Pro-TR Hybrids

The pro-TR hybrid (11.8 mg; 0.55 mg of TR mL^–1^) was dissolved in 2 mL of 100 mM sodium phosphate buffer, pH 8. 200 μL of 1.0 mg mL^–1^ native CT was added to PPH solution and the mixture was incubated at 37 °C. Aliquots of 40 μL were taken at 20, 40, 60, 90, and 120 min, diluted with 960 μL of deionized water (0.02 mg of TR mL^–1^ as final concentration), and then kept in an ice bath until activity assay was performed. The sample incubated for 120 min was filtered using a centrifugal filter MWCO 50 kDa, washed three times with 300 μL of deionized water, and freeze-dried. The chemical structure of the purified sample was confirmed by the ^1^H NMR spectrum, and the change in total molar mass was analyzed by SEC-MALLS.

### Activation of Pro-CT Hybrids

The Pro-CT hybrid (8.4 mg; 0.55 mg of CT mL^–1^) was dissolved in 2 mL of 100 mM Tris–HCl and 20 mM calcium chloride (pH 8). 200 μL of 0.2 mg mL^–1^ native TR was added to the PPH solution and the mixture was incubated at 37 °C. Aliquots of 100 μL were taken at 20, 40, 60, 90, and 120 min, diluted with 400 μL of deionized water (0.1 mg of CT mL^–1^ as final concentration), and then kept in an ice bath until activity assay was performed. The sample incubated for 120 min was filtered using a centrifugal filter MWCO 50 kDa, washed three times with 300 μL of deionized water, and freeze-dried. The chemical structure of the purified sample was confirmed by the ^1^H NMR spectrum, and the change in total molar mass was analyzed by SEC-MALLS.

### Exploring the Amplification Activity Cascade in a Dual Pro-Protease Hybrid System

A mixture of pro-CT hybrid (4.2 mg, 0.28 mg of CT mL^–1^) and pro-TR hybrid (5.9 mg, 0.28 mg of TR mL^–1^) in 100 mM Tris–HCl and 20 mM calcium chloride (1 mL, pH 8.0) solution were prepared. Native TR (100 μL, 0.2 mg mL^–1^) as a cascade activation trigger was added to the dual pro-protease hybrids solution. The mixture was incubated at 37 °C. At the selected time, an aliquot of the solution (2 μL) was taken from the mixture and added to the solution of BSA-polymer hybrid-based macrosubstrate (BSA-p(CBMA-*co*-MAOEG-AAPFpNA), 100 μL of 15.4 mg/mL in the mixture of deionized water and DMSO (50:50 vol %), 1.0 mM of pNA) in 100 mM sodium phosphate (898 μL, pH 8.0) in a cuvette. The rate of hydrolysis of the macrosubstrate was determined by recording the increase in absorbance at 412 nm. The change of enzymatic activity of the hybrid by cleaving of CT-inhibitor peptide moieties from the pro-CT hybrid was monitored by the comparison of the initial hydrolysis velocity of the macrosubstrate before adding a trigger protease to the solution of dual pro-protease hybrids.

## Results and Discussion

### Synthesis and Characterization of Pro-Protease Hybrids (Pro-TR and Pro-CT)

We designed artificial TR and CT zymogens based on PPH, which have a polymer backbone with cleavable side chains. For the synthesis of an artificial pro-TR hybrid, to ensure that it is activated by CT but not TR or EP, macromonomers containing a CT-cleavable phenylalanylalanine (Phe-Ala) peptide and a TR-inhibiting arginylproline (Arg-Pro) peptide (Figure 1A) were synthesized and modified with zwitterionic carboxybetaine methacrylate (CBMA), which provides excellent solubilization and protein stabilization properties. In contrast, the synthesis of the artificial pro-CT hybrid utilized a macromonomer containing an arginylglycine (Arg-Gly) peptide chain cleavable by TR and a CT-inhibiting phenylalanylsarcosine (Phe-Sar) peptide (Figure 1B)**.** In this case, the use of the unnatural amino acid sarcosine (*N*-methylglycine) as an inhibitory peptide demonstrates the breadth of possibilities in macromonomer design. The cleavable and inhibitory peptide sequences were based on the sequences of peptide substrates (Tos-Gly-Pro-Arg-pNA for TR and suc-Ala-Ala-Pro-Phe-pNA for CT) that show a high substrate affinity for TR or CT, respectively. Each cleavable and inhibitor peptide in the macromonomer is separated by a short PEG chain.

**Scheme 1:**
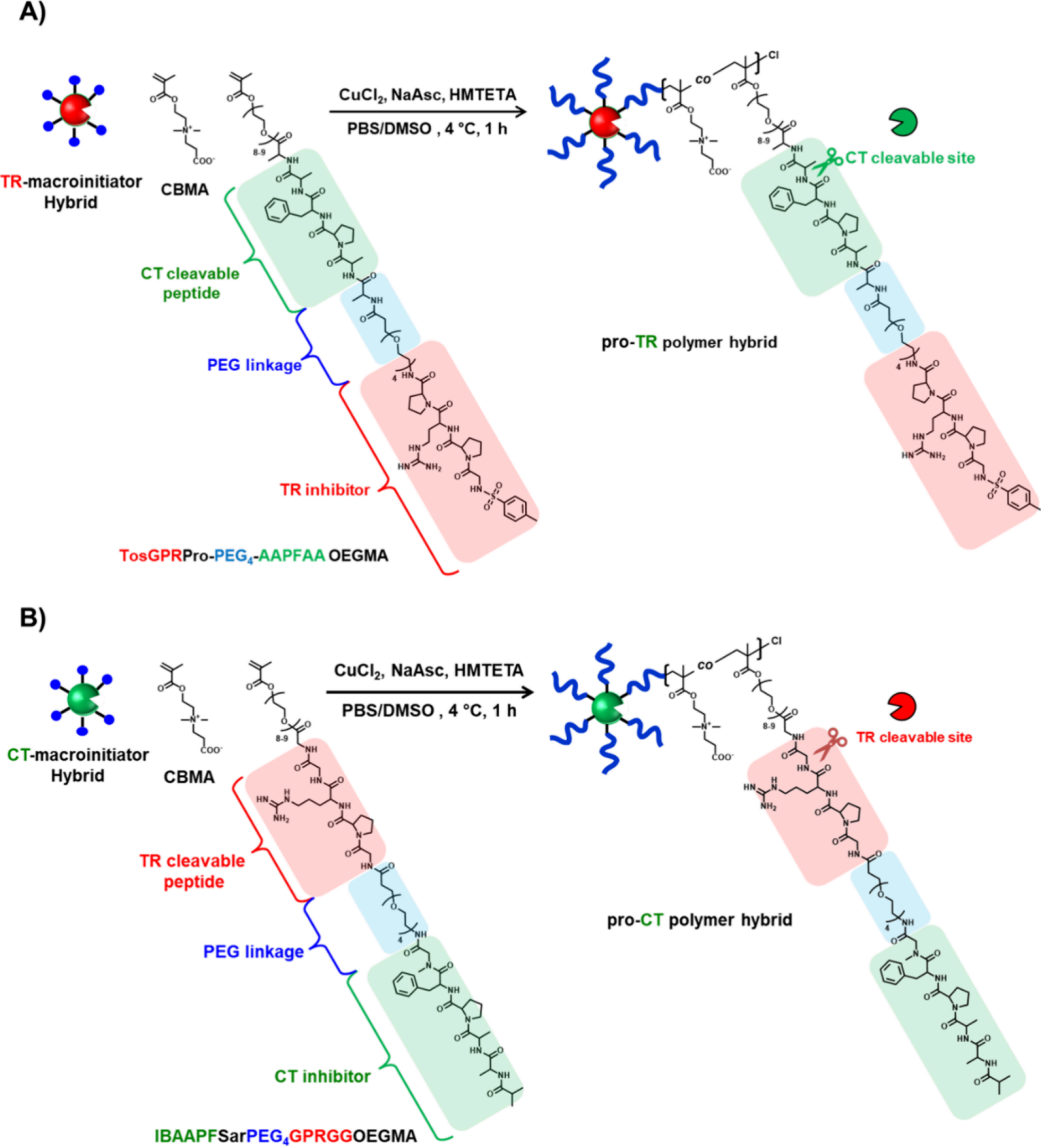
Preparation of Pro-Protease Hybrids by “Grafting-from” ATRP Approach An ATRP initiator was first reacted with amino groups on the CT and TR surfaces. Next, ATRP was used to graft copolymers of CBMA and macromonomers from the enzyme surface. (A) pro-TR hybrid with CT cleavable TR inhibitor, (B) pro-CT hybrid with TR cleavable CT inhibitor.

**Figure 2:**
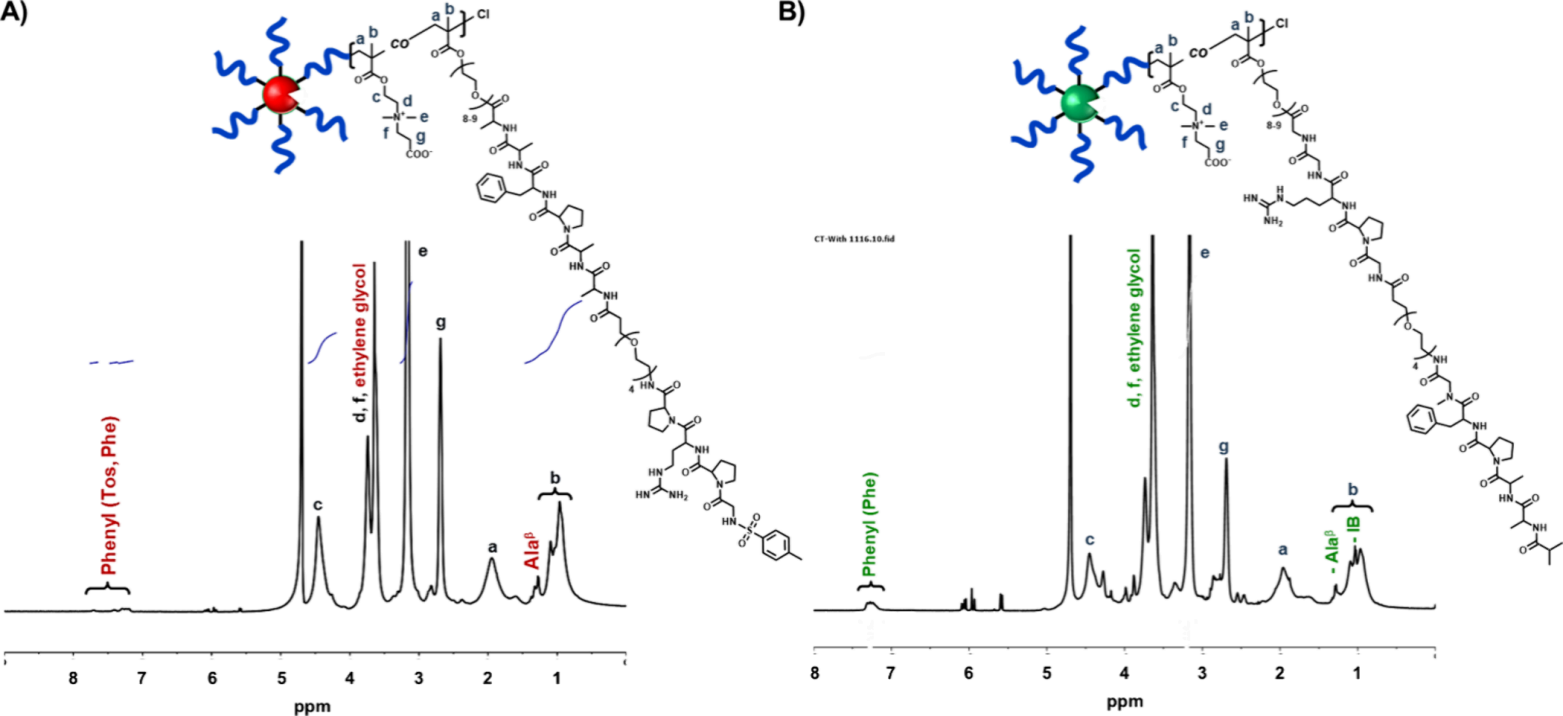
^1^H NMR spectra of pro-protease polymer hybrids. (A) ^1^H NMR spectrum of the pro-TR hybrid in D_2_O. (B) ^1^H NMR spectrum of pro-CT hybrid in D_2_O.

CT and TR macroinitiator hybrids were prepared by following established procedures, resulting in an average of 12 ATRP initiators per molecule for both CT and TR enzymes.^87^ Traditional ARGET-ATRP, involving the reduction of the copper-complex catalyst by sodium ascorbate, was carried out to synthesize an artificial pro-TR hybrid incorporating a TR inhibitor and a CT-cleavable peptide sequence in the polymer side chain (Scheme 1A). The macromonomer employed a PEG_4_ linker to clearly separate the inhibitor from the cleavable peptide moiety and to improve the solubility in aqueous polymerization solutions. Macromonomers containing glycine or PEG_2_ as linkers exhibited reduced solubility in the aqueous polymerization solution, leading to the introduction of fewer inhibitor moieties into the hybrids and resulting in less loss of activity (Figure S15 and Table S3). Similarly, a pro-CT hybrid (pro-CT) was prepared (Scheme 1B), featuring a CT inhibitor and TR cleavable peptide sequence in the polymer side chain. As controls without the inhibitory domain, poly(CBMA) was exclusively grafted from both CT and TR, resulting in CT-pCBMA and TR-pCBMA, respectively. The small-molecule copper-complex catalyst and unreacted monomers were easily removed by dialysis, and these PPHs were then purified. The chemical structure of pro-CT and pro-TR hybrids was determined by ^1^H NMR spectra in D_2_O (Figure 2A, B), which showed proton signals from the comonomer CBMA (Figure 2A, B, a–g) and specific ethylene glycol and peptide side chain proton signals from the macromonomer.

The absolute total molar masses and the dispersity of the synthesized pro-protease hybrids were estimated by SEC-MALLS, and the average polymerization degrees of CBMA and macromonomer per polymer chain were estimated from the total molecular mass and the proportions estimated by ^1^H NMR of artificial pro-TR and pro-CT hybrids (Table 1, entries 1 and 2, entry 4, respectively). The number of macromonomers per polymer chain for the pro-TR and pro-CT hybrids is 2.6 and 2.8, resulting in 31 or 33 inhibitor peptides per hybrid molecule, respectively.

**Table 1:** Characterization and Enzymatic Activity of Inhibited and Activated Pro-TR Polymer Hybrid^a^

		SEC-MALLS analysis				Michaelis–Menten parameters^d^		
		*M*_n_^b^	*M*_w_^b^	*Đ*^b^	DP^c^	*K*_M_	*k*_cat_	*k*_cat_/*K*_M_
entry	samples	kg mol^–1^	kg mol^–1^		(CBMA/inhibitor)	μM	s^–1^	μM^–1^ s^–1^
	native TR					22.2 ± 1.9	63.5 ± 1.3	2.87 ± 0.25
1	pro-TR hybrid	246.2	338.9	1.38	60.4/2.6	26.2 ± 1.2	23.2 ± 0.3	0.87 ± 0.09
2	activated TR hybrid	187.7	264.7	1.41	59.2/0.8	16.3 ± 0.6	32.1 ± 0.5	1.97 ± 0.04
3	TR-pCBMA hybrid	272.8	492.0	1.80	99.2/--	18.8 ± 2.8	45.7 ± 1.1	2.43 ± 0.36

a Polymerization conditions: [M]_0_:[I]_0_:[CuBr_2_]_0_:[HMTETA]_0_:[NaAsc]_0_ = 50:0.5:5.0:0.5:6.0 (mM) in PBS and 10 vol % DMSO. Four °C, 1 h. [M]; [CBMA] only or [CBMA]:[Macromonomer-inhibitor] = 9:1 molar ratio.

b Determined by SEC-MALLS.

c Determined by SEC-MALLS and ^1^H NMR spectra.

d Conditions: [TR]_0_ = 8.4 nM, [Tos-GPR-pNA]_0_ = 0–302 μM, buffer 100 mM Tris–HCl and 20 mM CaCl_2_ (pH 8.0) at 37 °C.

**Table 2:** Characterization and Enzymatic Activity of Inhibited and Activated Pro-CT Polymer Hybrid^a^

		SEC-MALLS analysis				Michaelis–Menten parameters^d^		
		*M*_n_^b^	*M*_w_^b^	*Đ*^b^	DP^c^	*K*_M_	*k*_cat_	*k*_cat_/*K*_M_
entry	samples	kg mol^–1^	kg mol^–1^		(CBMA/inhibitor)	μM	s^–1^	μM^–1^ s^–1^
	native CT					90.1 ± 12.5	43.6 ± 1.5	0.484 ± 0.069
4	pro-CT hybrid	191.2	294.3	1.54	39.7/2.8	165.6 ± 34.5	5.6 ± 0.4	0.034 ± 0.001
5	activated CT hybrid	137.7	198.3	1.44	38.0/0.7	64.0 ± 11.0	6.0 ± 0.2	0.093 ± 0.002
6	CT-pCBMA hybrid	114.2	219.6	1.52	32.5/--	84.2 ± 13.3	24.6 ± 1.0	0.292 ± 0.022

a Polymerization conditions: [M]_0_:[I]_0_:[CuBr_2_]_0_:[HMTETA]_0_:[NaAsc]_0_ = 50:0.5:5.0:0.5:6.0 (mM) in PBS and 10 vol % DMSO. Four °C, 1 h. [M]; [CBMA] only or [CBMA]:[Macromonomer-inhibitor] = 9:1 molar ratio.

b Determined by SEC-MALLS.

c Determined by SEC-MALLS and ^1^H NMR spectra.

d Conditions: [CT]_0_ = 40 nM, [suc-AAPFpNA]_0_ = 0–960 μM, buffer 100 mM sodium phosphate (pH 8.0) at 37 °C.

As shown in Tables 1 (entry 1) and 2 (entry 4), the enzymatic activities of pro-TR and pro-CT hybrids were compared with those of native proteases using small peptide substrates (Tos-Gly-Pro-Arg-pNA for TR and suc-Ala-Ala-Pro-Phe-pNA for CT, respectively). Compared with the Michaelis constant of native TR for the peptide substrate (*K*_M_ = 22.2 μM), the apparent *K*_M_ value of the pro-TR hybrid (*K*_M_ = 26.2 μM) was similar. However, the turnover value of the pro-TR hybrid (*k*_cat_ = 23.2 s^–1^) was approximately three times lower than that of the native TR (*k*_cat_ = 63.5 s^–1^), resulting in the apparent catalytic efficiency remaining at only about 30%. In contrast, compared with the Michaelis constant of native CT for the peptide substrate (*K*_M_ = 90.1 μM), the apparent *K*_M_ value of the pro-CT hybrid (*K*_M_ = 165.6 μM) was 1.8 times higher, indicating a decreased affinity for the peptide substrate. The apparent turnover value of the pro-CT hybrid (*k*_cat_ = 5.6 s^–1^) was approximately 8-fold lower than that of the native CT (*k*_cat_ = 43.6 s^–1^), and thus the apparent catalytic efficiency of the pro-CT hybrid *(k*_cat_/*K*_M_ = 0.034 μM^–1^ s^–1^) was only about 10% compared to that of the native CT (0.484 μM^–1^ s^–1^).

Our hypothesis was that incorporating inhibitory peptides into the polymer backbone of pro-TR and pro-CT would significantly reduce their catalytic activity and render them inactive; however, the synthesized artificial pro-proteases were not completely inactive like the natural zymogens. To deliver the polymer-anchored inhibitor peptides to the active site of each protease hybrid, this report employed a strategy of functionalizing as many polymer chains as possible on the surfaces of TR and CT. In TR, 12 of the 15 amino groups (*N*-terminus and 14 Lys) are modified by the ATRP initiator, and it is estimated that at least three amino groups, including the *N*-terminus Ile^16^ and 7 Lys (K^60^, K^97^, K^175^, K^188^, K^222^, K^224^, and K^230^) near the active site, are modified by the ATRP initiator (Figure S20A). In CT, 6 Lys (K^87^, K^90^, K^93^, K^107^, K^175^, and K^177^) are located near the active site, but K^90^ and K^177^ are only modified with the ATRP initiator (Figure S20B).^61,84^ To verify the inhibitory ability of the inhibitory peptides used in the PPH-based pro-protease hybrids examined in this study, we calculated the *K*_i_ values of the inhibitory peptide sites and the macromonomers containing a cleavable inhibitor peptide group and compared their affinity for the natural enzyme with the respective peptide substrates. The *K*_i_ values of the inhibitory peptide site (Tos-Gly-Pro-Arg-Pro-NHBzl) and the macromonomer (TosGPRProPEG_4_AAPFOEGMA) for TR were 430 and 150 μM (Figure S9), respectively, which were approximately 20- and 7.5-fold larger than the *K*_M_ (20 μM) of the peptide substrate (Tos-Gly-Pro-Arg-pNA), indicating a small affinity of the inhibitory peptide site to the native TR. The *K*_i_ value of the inhibitor peptide site (isobutyryl-Ala-Ala-Pro-Phe-Sar-OMe) and the macromonomer (IBAAPFSarPEG_4_GPRGGOEGMA) against CT were 660 and 260 μM (Figure S12), respectively, which were 7- and 2.8-fold higher than the *K*_M_ of suc-Ala-Ala-Pro-Phe-pNA (90 μM), indicating that the inhibitory effect of the inhibitor peptide against CT was limited. The affinity of each macromonomer used in this study for the native protease was smaller than that of the peptide substrate, and even though the localized concentration of the inhibitor peptide on the protease was high due to immobilization on the polymer backbone, the synthesized artificial pro-protease hybrids were not completely inactive like the natural zymogens. Furthermore, it is also possible that the flexibility and the extended structure of the hydrophilic zwitterionic pCBMA prevent the inhibitor peptide from effectively reaching the active site.

To render artificial pro-protease hybrids based on PPH completely inactive, it will be necessary to improve the affinity of the inhibitor peptide to be immobilized onto the polymer backbone and further refine the PPH architecture by using multiheaded ATRP initiators or hyperbranched polymer strategies to increase the density of the polymer chains. However, if there is a high density of polymer chains near the active site, the steric hindrance and exclusion effect between the polymer chains may prevent the reach of the inhibitor peptide to the active site. It may be ideal to have a single polymer modified near the active site so that the anchor inhibitor peptide can effectively access the active site.

### Activation of Pro-TR and Pro-CT Hybrids

**Scheme 2:**
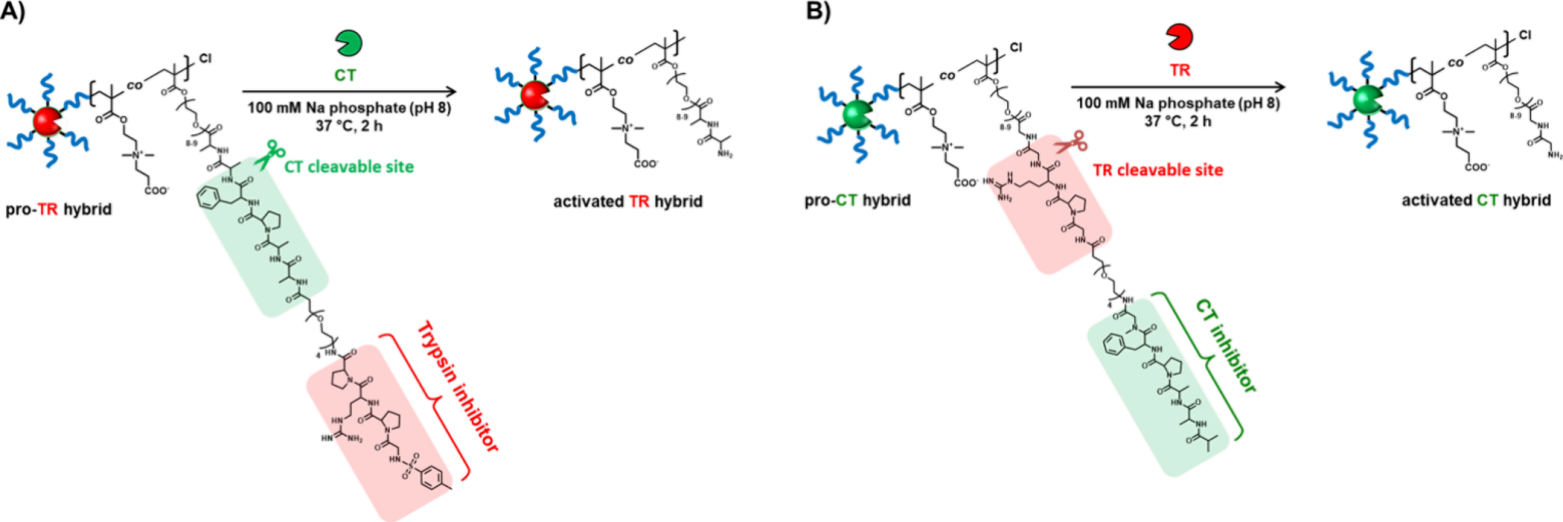
Activation (Cleavage of Inhibitor) of Pro-Protease Hybrids by Trigger Protease (A) Activation of pro-TR hybrid by native CT. (B) Activation of pro-CT hybrid by native TR.

**Figure 3:**
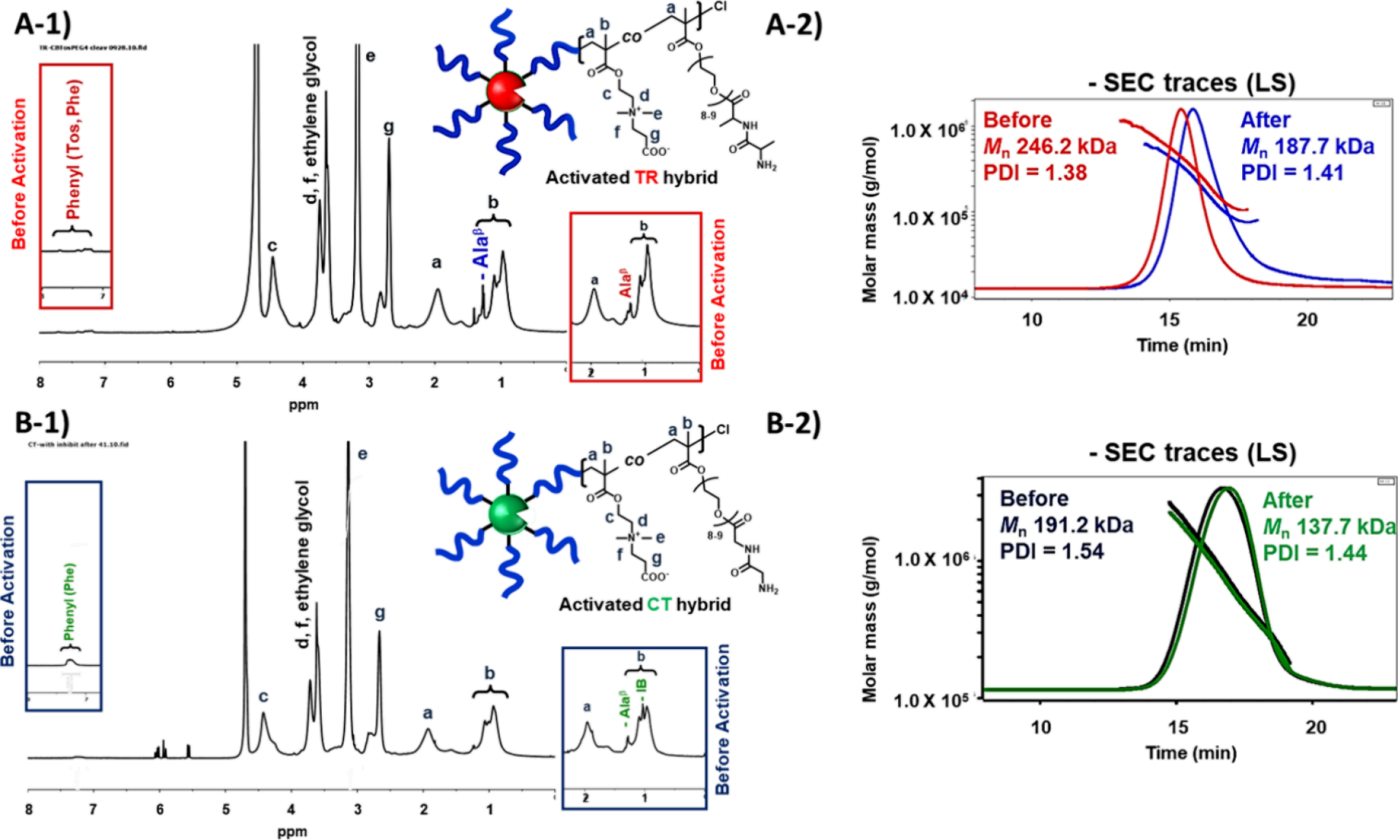
^1^H NMR and SEC traces of pro-protease biohybrids before and after activation. (A-1) ^1^H NMR spectrum of the activated pro-TR polymer hybrid by native CT. The red boxes show the proton signals of the hybrid before activation. (A-2) SEC trace of pro-TR hybrid before and after incubation with native CT. (B-1) ^1^H NMR analysis of the activated pro-CT polymer hybrid by native TR. The green boxes show the proton signals of the hybrid before activation. (B-2) SEC trace of the pro-CT hybrid before and after incubating with native TR.

The preparation of pro-protease hybrids based on PPH was attempted by using modified polymers containing inhibitor peptides. This involved the effective removal of the inhibitor site from the hybrid. The activation of the pro-protease hybrids was explored by cleaving the inhibitory peptides incorporated into the modified polymers with exogenous proteases. While native trypsinogen is activated by enteropeptidase and TR in nature, we designed the artificial pro-TR hybrid to be activated by CT. As shown in Scheme 2A, incubation with native CT leads to specific hydrolysis at the amide bond on the carboxyl side of Phe, and the resulting TR inhibitory peptide is cleaved from the pro-TR hybrid. The pro-TR hybrid was treated in a buffer solution (pH 8) at 37 °C for 2 h in the presence of native CT, and then the hybrid was purified by dialysis and characterized by ^1^H NMR analysis and SEC-MALLS (Table 1, entry 2 and Figure 3A-1, A-2). In the ^1^H NMR spectrum of the purified activated TR hybrid, the proton signals of the polymer backbone derived from CBMA were observed, but the phenyl proton signals of phenyl groups from Phe in the CT cleavage site and from Tos of the TR inhibitor peptide were greatly reduced (Figure 3A-1). SEC-MALLS analysis for estimating the absolute total molar masses of the hybrids before and after CT incubation showed that the SEC trace of the activated TR hybrid uniformly shifted to the lower molar mass region (Figure 3A-2)**,** resulting in a decrease in total molar mass from 246.2 to 187.7 kg/mol (Table 1, entries 1 and 2)**.** Estimates from the molar mass decrease and ^1^H NMR analysis indicate that approximately 22 of the average 31 inhibitory peptides on the pro-TR hybrid were cleaved off and removed by CT incubation. Similarly, the incubation of native TR with the pro-CT hybrid led to specific hydrolysis at the amide bond on the carboxyl side of Arg, cleaving the CT inhibitory peptide to generate the activated CT hybrid. The resulting activated CT hybrids were characterized by SEC-MALLS and ^1^H NMR analysis (Table 2, entry 5, Figure 3B-1, B-2). In the ^1^H NMR spectrum of the activated CT hybrids, proton signals from the polymer backbone derived from CBMA were mainly observed, while the proton signals from the phenyl group of Phe, the methyl side chain of Ala, and the isobutyryl terminus in the CT inhibitory peptide were significantly reduced (Figure 3B-1). SEC-MALLS analysis of the activated CT hybrid showed that its SEC trace shifted to a lower molar mass region without any change in shape compared to the pro-CT hybrid (Figure 3B-2), and the total molar mass also decreased from 191.2 to 137.7 kg/mol (Table 2, entries 4 and 5). TR incubation also cleaved the average 25 of 33 CT inhibitor peptides from the pro-CT hybrid. Characterization by SEC-MALLS and ^1^H NMR analysis demonstrated that the inhibitory peptide sites could be removed from the artificial pro-protease by the corresponding protease.

**Figure 4:**
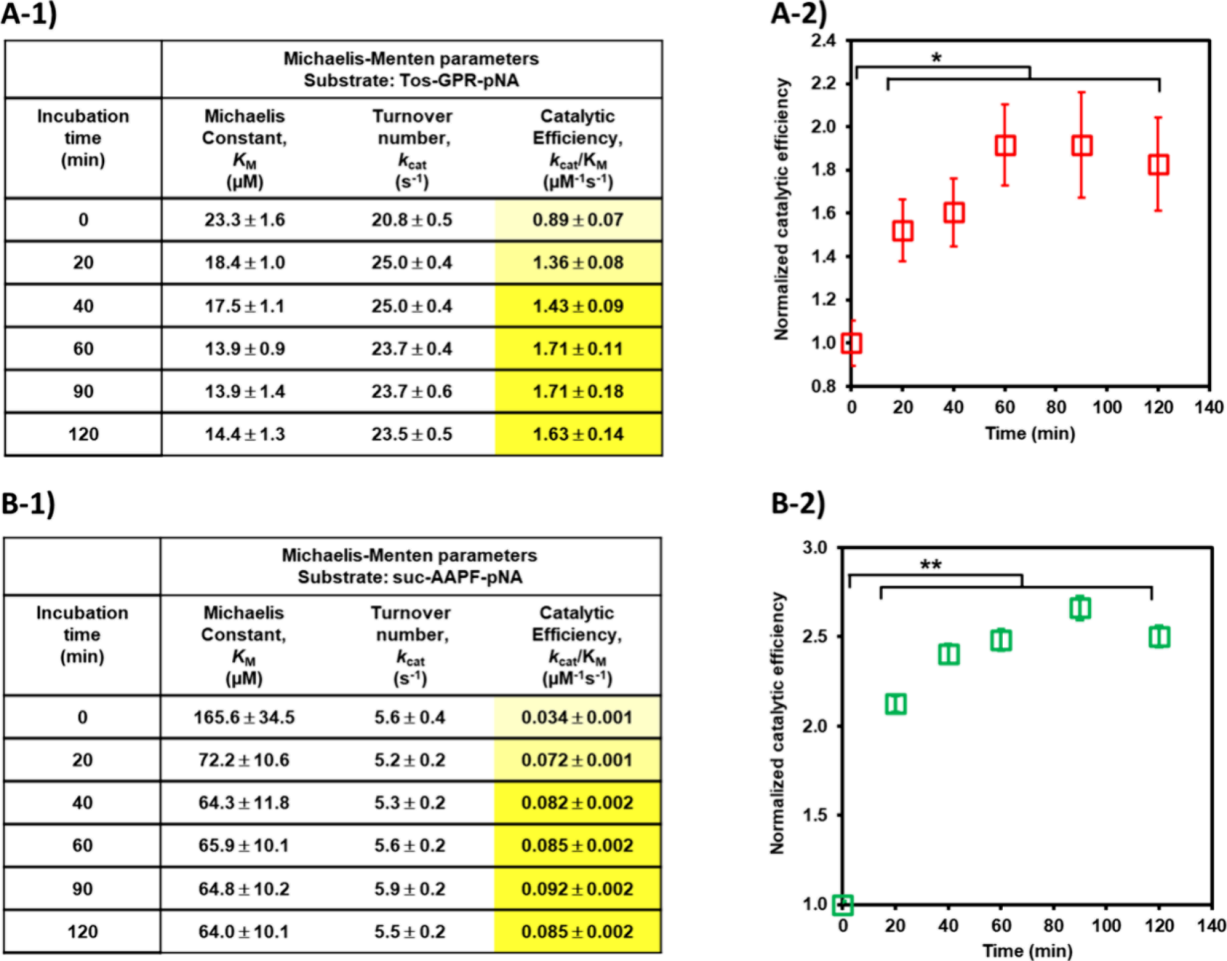
Relative enzymatic activity of pro-protease polymer hybrids before and after adding trigger protease. (A-1) Michaelis–Menten parameters toward Tos-Gly-Pro-Arg-pNA for pro-TR hybrid activated by native CT incubation. (A-2) Relative enzymatic efficiency of pro-TR hybrid before and after incubating with native CT. (B-1) Michaelis–Menten parameters toward Suc-Ala-Ala-Pro-Phe-pNA for pro-CT hybrid activated by native TR incubation. (B-2) Relative enzymatic efficiency of pro-CT hybrid before and after incubating with native TR. Data are presented as the mean ± standard deviation (SD) of three experiments. Mean differences between experimental groups were tested with an unpaired *t* test. Values were significantly different at the *p < 0.05 or **p < 0.01.

Next, we sought to observe the enhancement of enzyme activity upon transition from synthetic pro-protease hybrids to active protease hybrids. The active protease hybrid, in which the inhibitor peptide is cleaved, should recover its catalytic performance, retaining the bound polymer backbone on its surface. First, we monitored the time course of enzyme activity of the inactive pro-TR hybrid treated with native CT using peptide substrates, Tos-Gly-Pro-Arg-pNA (Figure 4A-1, A-2)**.** Aliquots of the treated TR hybrid solution were taken at designated times and used to estimate the Michaelis–Menten parameters without purification. After 20 min of CT treatment, there was a slight increase in the affinity and turnover of the TR hybrid for the peptide substrate, resulting in a 1.5-fold increase in catalytic efficiency (Figure 4A-1)**.** Up to 60 min after treatment, the catalytic efficiency of the activated CT hybrid was nearly doubled compared to that of the untreated pro-CT hybrid. After 60 min, no further improvement in catalytic activity was observed, and the catalytic efficiency of the activated TR hybrid reached a plateau (Figure 4A-2). Removal of the peptide inhibitor from the side chain improved the enzymatic activity of pro-TR, restoring the catalytic efficiency of the activated TR hybrid (*k*_cat_/*K*_M_ = 1.97 μM^–1^ s^–1^, Table 1, entry 2) to approximately 80% of that of TR-pCBMA (2.43 μM^–1^ s^–1^, Table 1, entry 3), a model hybrid consisting of the pCBMA backbone without the peptide inhibitor in the side chain. Because the cleavable peptide sequence on the synthetic pro-CT hybrid was sensitive to the native TR enzyme, we incubated it with trigger TR to form an activated CT hybrid and observed the change in enzyme activity using a small peptide substrate (suc-Ala-Ala-Pro-Phe-pNA) (Figures 4B-1, B-2). As with the pro-TR hybrids, aliquots of samples were taken at 20–30 min intervals and analyzed for catalytic performance without purification. The TR-treated pro-CT hybrid did not show enhanced turnover, but compared to the untreated hybrid, the *K*_M_ value for the peptide substrate decreased from 165.6 to 72.2 μM in 20 min, resulting in an approximately 2-fold increase in enzyme efficiency (Figure 4B-1)**.** Furthermore, a gradual decrease in *K*_M_ value was observed until 90 min of incubation, and the normalized catalytic efficiency increased up to 2.5-fold before plateauing (Figure 4B-2). In the case of activation of the pro-CT hybrid, the catalytic efficiency of the activated CT hybrid (*k*_cat_/*K*_M_ = 0.093 μM^–1^ s^–1^, Table 2, entry 5) was only about 30% of that of the model TR-pCBMA hybrid (0.292 μM^–1^ s^–1^, Table 2, entry 6). Unlike the activation of a completely inactive zymogen, the transition from a synthetic Pro-protease to an active hybrid may give the impression of a small activity gain, but the presence of cleaved inhibitory peptides in the environment must be taken into account when investigating the enzymatic activity of activated TR and CT polymer hybrids. For the activation of pro-protease hybrids by the trigger enzyme performed in this study, it was calculated that 1.6 mM inhibitor peptide was present in the experimental solution for both pro-TR and pro-CT hybrids, respectively (Supporting Information). After activation, the concentrations of released inhibitor peptides were estimated to be 1.1 and 1.2 mM for TR and CT hybrids, respectively. These concentrations are sufficiently large compared to the inhibition constants of inhibitor peptides and macromonomers for the respective enzymes to sufficiently affect the enzymatic assay of the activated hybrids and explain the spurious activation of the pro-protease hybrids. Furthermore, densely grafted zwitterionic pCBMA hinders access of the trigger enzyme to cleavable inhibitor peptides, especially those close to the protein surface. As a result, the removal of all inhibitor peptides was incomplete, leaving approximately 8 and 9 inhibitor peptides in the active TR and CT hybrids, respectively (Tables 1 and 2). This is also likely the cause of the incomplete activation.

### Amplified Activation through Cascade Activity of Artificial Zymogens (Pro-CT and Pro-TR)

**Scheme 3:**
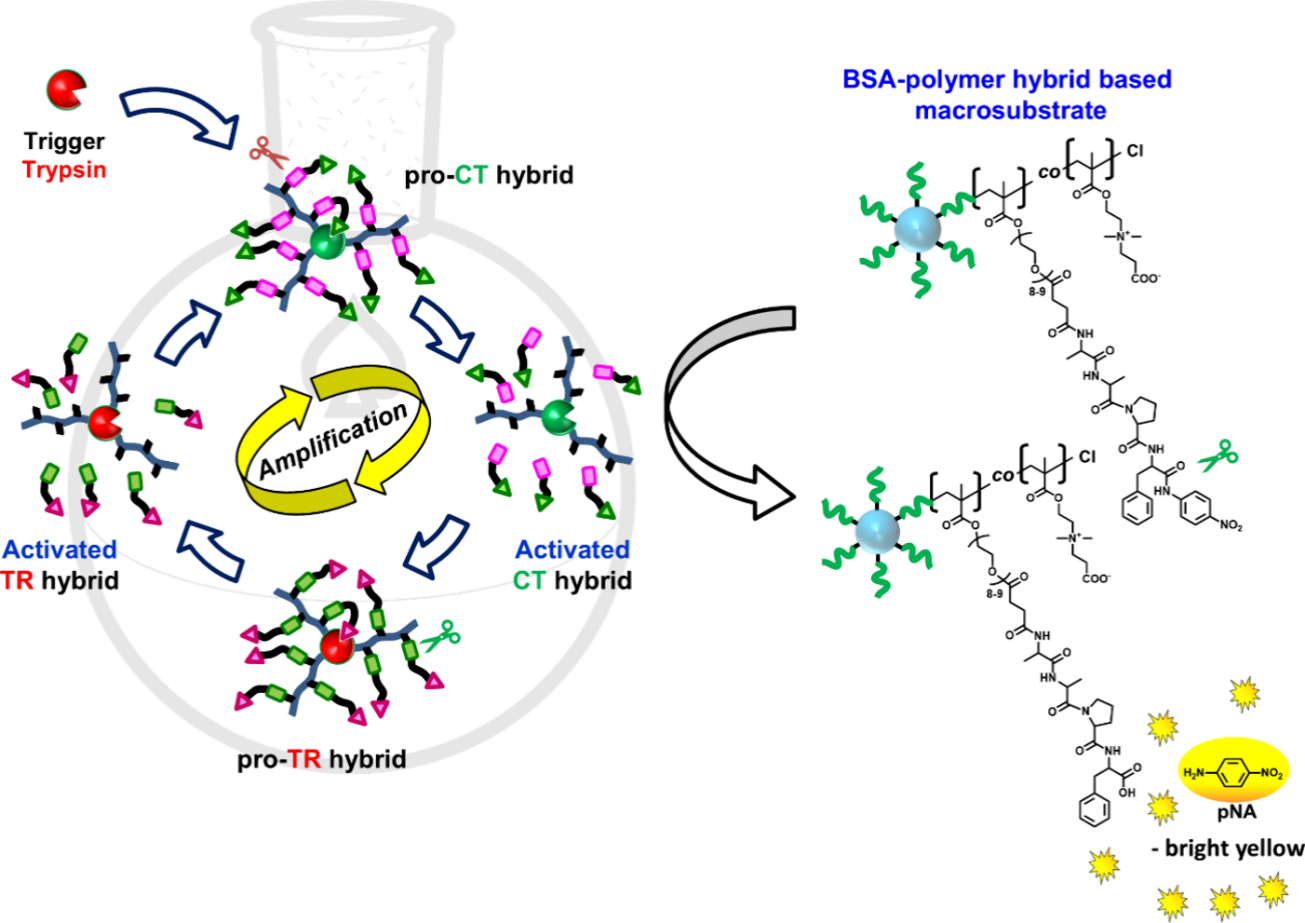
Activation Amplification through Cascade Activity of Artificial Pro-CT and Pro-TR Hybrids by Trypsin Incubation The red scissors indicate the site of cleavage by activated TR, and the green scissors indicate the site of cleavage by activated CT, respectively.

**Figure 5:**
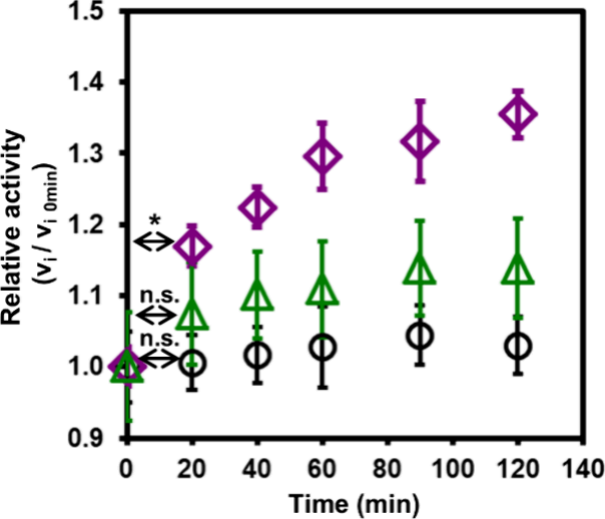
Increment of relative enzymatic activity of the dual pro-CT and pro-TR hybrid cascade system and single pro-CT hybrid using BSA-macrosubstrate. Dual pro-TR and pro-CT hybrids without native TR trigger treatment (open black circle), single pro-CT hybrid with native TR trigger treatment (open green triangle), and dual pro-TR and pro-CT hybrids with native TR trigger treatment (open purple diamond). Data are presented as the mean ± standard deviation (SD) of three experiments. Mean differences between experimental groups were tested with an unpaired *t* test. Values were significantly different at the *p < 0.05 or n.s. = not significant.

We demonstrated that synthetic pro-protease hybrids prepared by PPH using TR and CT could reduce apparent enzyme activity by conjugating a cleavable inhibitor peptide and restore enzyme activity by cleavage with an exogenous protease. In nature, a cascade reaction is formed in which trypsinogen is activated by enteropeptidase or active TR, and chymotrypsinogen is activated by these activated TRs. However, the pro-TR hybrid designed in this study is activated by active CT through the modification of a cleavable TR inhibitor peptide that is sensitive to CT. Cascade reactions are characterized by a domino effect in which each successive reaction is contingent upon the chemical functionality generated in the preceding step.^102^ These reactions are useful for amplification mechanisms in response to even a weak environmental trigger for the activation of an enzyme.^103^ The prepared activated forms of the pro-protease hybrid can activate their inactive counterparts, allowing the construction of an activity amplification cascade using dual pro-protease hybrids triggered by an exogenous protease. To verify the activity amplification cascade driven by the trigger protease, we used the synthetic artificial pro-TR and pro-CT hybrids discussed in the previous section. The relative activity increment of the dual pro-protease activity amplification cascade system was evaluated in comparison with that of a single pro-CT hybrid using bovine serum albumin (BSA)-polymer hybrid-based macrosubstrates (Scheme 3). The advantage of using the BSA-based macrosubstrate immobilized by the polymer chain with multiple peptide substrates on the BSA surface is that the size of the macrosubstrate is much larger than that of small peptide substrates. This significantly reduces the penetration of the PPH-based artificial pro-protease hybrid into the polymer layer, resulting in a lower background colorimetric signal.^87^ As shown in Figure 5, the change behavior of the relative activity of the single pro-CT hybrid and the dual pro-TR and pro-CT hybrid mixture before and after treatment with the trigger TR was observed. The relative activity of a single pro-CT hybrid increased only by 7% in 20 min with TR incubation, followed by a slow increase in activity, and after 1 h, their relative activity reached 1.1-fold compared to values before TR treatment (Figure 5, open green triangle). In the case of the dual pro-protease hybrid mixture, a rapid activity increase of 17% was observed after 20 min compared to a single pro-CT, and the activity reached 1.3-fold after 1 h (Figure 5, open purple diamond), thus successfully proving the construction of an activity amplification cascade with PPH based dual artificial pro-protease hybrids. In the control experiment performed in the absence of native TR in the solution in which the two hybrids were mixed, no activation of pro-CT occurred and therefore no increase in relative activity in the system could be observed, proving that the artificial pro-proteases do not interfere with each other’s activation (Figure 5, open black circle). These results underline the critical role of the trigger protease in activating the cascade and confirm the amplification of the enzymatic activity when a dual artificial zymogen system is applied. Using the BSA-polymer hybrid-based macrosubstrate, a slight increase in activity was observed compared with the activity with the peptide substrate. This is because the activated hybrid, from which the inhibitor peptide in the side chain of the modified polymer was removed, still carried the remaining polymer backbones. These backbones hindered penetration of the large BSA macrosubstrate. Additionally, the activated hybrids that retained the polymer backbones limited the cleavage of the inhibitory peptide of the counterpart artificial pro-protease hybrid, causing a delayed increase in activity. Therefore, future PPH-based artificial pro-protease hybrids will need to be modified to remove not only the inhibitor peptide side chain but also the polymer backbone itself to achieve a more significant activity amplification effect. Furthermore, in the use of dual or multiple protease cascade systems, the activated proteases may digest each other or decrease concentration due to autolysis, so a rapid activation process and short-term detection of activated proteases are required.

## Conclusion

In conclusion, taking inspiration from nature, we designed artificial zymogens based on protein–polymer hybrids (PPHs) and synthesized pro-protease hybrids using commercially available trypsin (TR) and chymotrypsin (CT) by utilizing the surface-initiated ATRP technique to modify polymer chains bearing abundant cleavable inhibitor peptides on the respective protease surfaces. Interestingly, contrary to nature, an artificial pro-TR hybrid was synthesized that can be activated by active CT. These artificial pro-protease hybrids could not be rendered completely inactive like zymogen, but upon exposure to specific complementary proteases, the modified inhibitory peptides were sequentially cleaved from the hybrids, enhancing the enzymatic activity of the hybrids. Furthermore, since pro-TR and pro-CT hybrids can activate each other, a dual pro-protease hybrid system was conceived and implemented that can amplify activity through an enzyme activation cascade in response to trigger proteases in the environment. Although there is room for improvement, the advantage of PPH-based artificial zymogen hybrids is the infinite choice of diverse core enzymes, cleavage and inhibition site peptides, and polymers with various functionalities depending on the intended application, utilizing a controlled radical polymerization technique. Further advances in the artificial zymogen strategy are therefore expected to allow proteins to be stored in an inactive state and activated in response to specific environmental stimuli, and this unique precision-driven approach could open new avenues for more sophisticated medical applications and biodetection platforms.

## Supporting Information Available

It contains instrumentation preparation and characterization of macromonomers containing cleavable and inhibitory peptides (Tos-Gly-Pro-Arg-Pro-PEG_4_-Ala-Ala-Pro-Phe-Ala-Ala-OEGMA and IB-Ala-Ala-Pro-Phe-Sar-PEG_4_-Gly-Pro-Arg-Gly-OEGMA), and results and procedures for inhibition assays of the inhibitor peptide moieties used in the hybrids (PDF)
