# Supplementary material for: Artificial Zymogen Based on Protein–Polymer Hybrids
Source: Biomacromolecules. 2024 Oct 18;25(11):7433–45. doi: 10.1021/acs.biomac.4c01079 (PMC11558679; doi:10.1021/acs.biomac.4c01079)
Supplement: Supplementary file 1 — bm4c01079_si_001.pdf [file bm4c01079_si_001.pdf]

Supplementary Information

Artificial Zymogen Based on Protein-Polymer Hybrids

Hironobu Murata[1‡], Kriti Kapil[1‡], Bibifatima Kaubayeva,[1], Alan J. Russell[2], Jonathan S. Dordick[3] Krzysztof Matyjaszewski[1]*

[1] Department of Chemistry, Carnegie Mellon University, 4400 Fifth Avenue, Pittsburgh, PA 15213, United States.

[2]Amgen Research, 1 Amgen Center Drive, Thousand Oaks, California 91320, United States

[3] Department of Chemical and Biological Engineering, Center for Biotechnology & Interdisciplinary Studies, Rensselaer Polytechnic Institute, Troy, NY 12180, United States

[‡]These authors contributed equally.

Supporting Experimental Section

Instrumentation and Sample Preparation. ^{1}H NMR spectra were recorded on a spectrometer (Bruker Avance™ III 500 MHz NMR Instrument) in the NMR facility located in Center for Molecular Analysis, Carnegie Mellon University, Pittsburgh, PA, with deuterium oxide (D_2O), DMSO-d_6 and C*D*Cl_3. UV−VIS spectra were obtained and used for protein concentration determination using a UV−VIS spectrometer (Lambda 2, PerkinElmer). SEC-MALS characterizations were performed using Agilent SEC system equipped with Waters Ultrahydrogel Linear column, using Dulbecco's Phosphate Buffered Saline, DAWN HELEOS-II (Wyatt) MALS detector and Optilab T-rEX (Wyatt) RI detector in located at Carnegie Mellon University.

Synthesis of macromonomer containing TR inhibitor and CT cleavable peptide moieties (Tos-Gly-Pro-Arg-Pro-PEG$_4$-Ala-Ala-Pro-Phe-Ala-Ala-OEGMA). A macromonomer containing a trypsin inhibitor and a chymotrypsin-cleavable peptide moieties was synthesized

using the procedure shown below (**Scheme S1**), with reference to a previously reported method for synthesizing macromonomers containing an oligopeptide using a solution phase method (**Scheme S1**).[1-3]

Scheme S1. Synthetic scheme for macromonomer (Tos-Gly-Pro-Arg-Pro-PEG$_4$-Ala-Ala-Pro-Phe-Ala-Ala-OEGMA) containing trypsin inhibitor and chymotrypsin cleavable peptide groups.

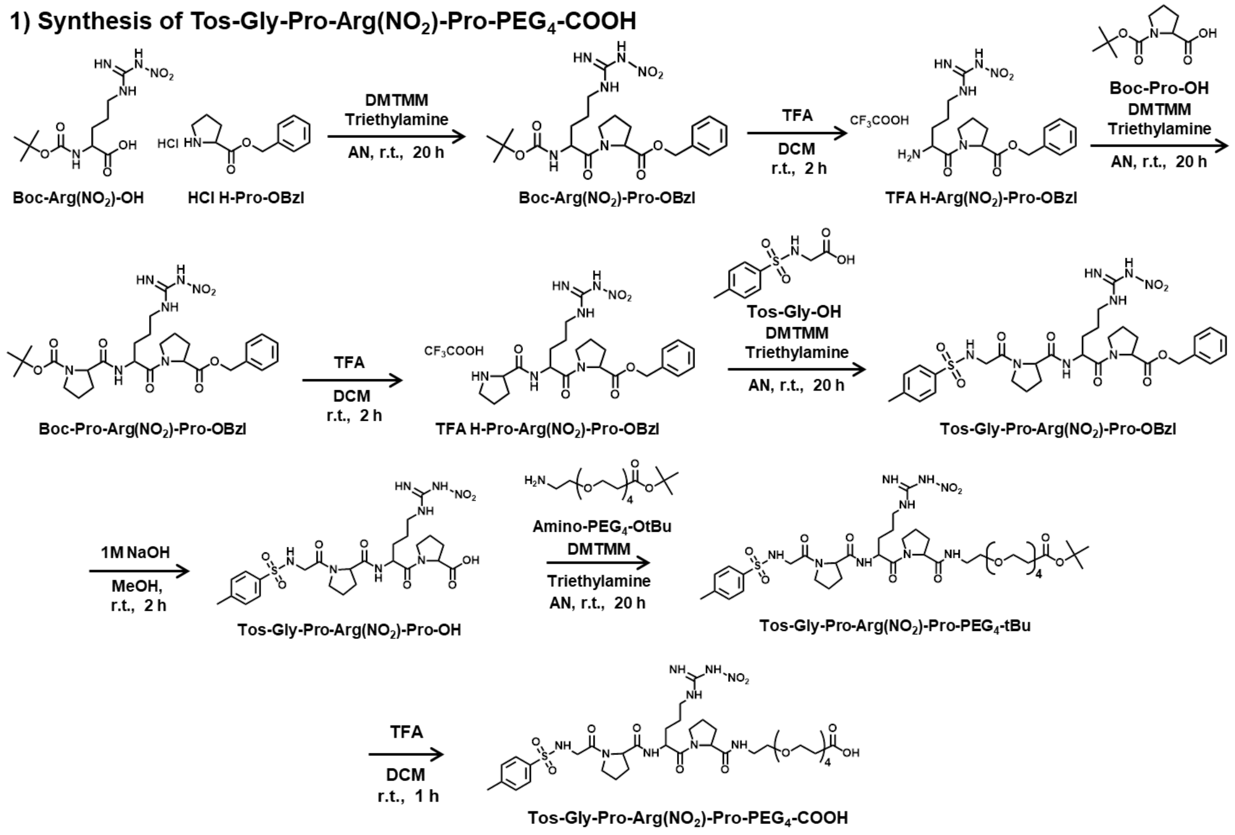

2) Synthesis of HCl H-Ala-Ala-Pro-Phe-OBzl

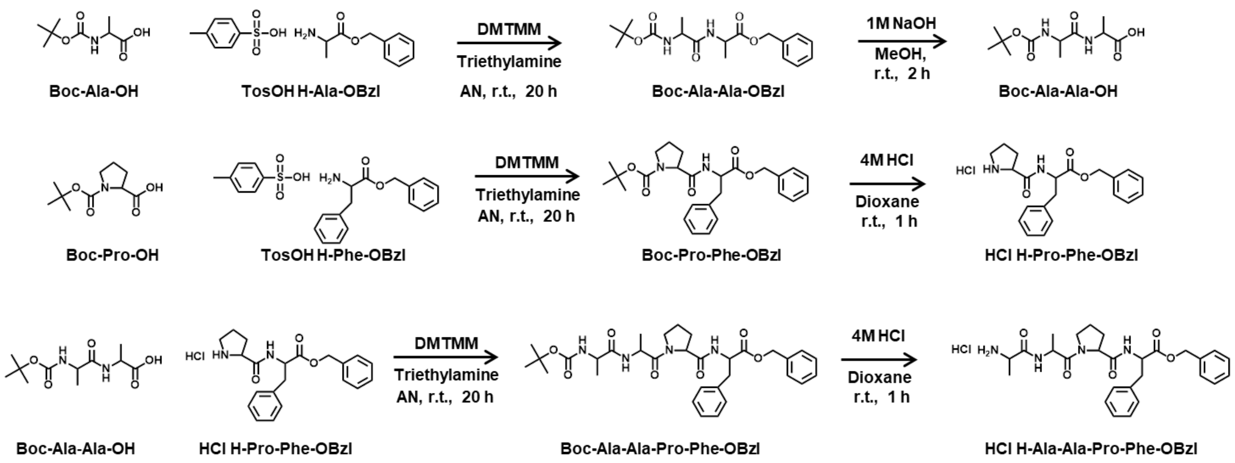

3) Synthesis of HCl H-Ala-Ala-OEGMA

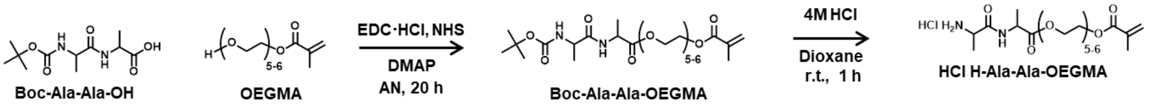

4) Synthesis of macromonomer containing TR-inhibitor and CT cleavable peptide group

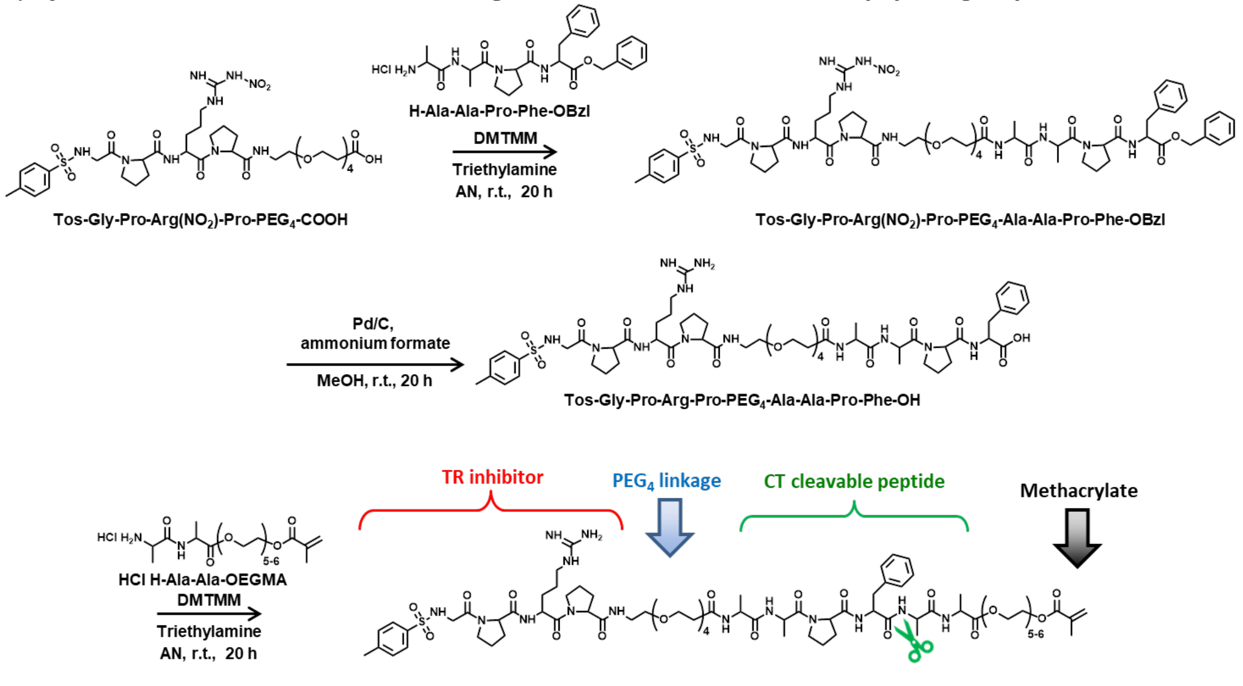

1) Synthesis of Tos-Gly-Pro-Arg(NO$_2$)-Pro-PEG$_4$-COOH. A solution of Boc-Arg(NO$_2$)-OH (6.4 g, 20 mmol) and DMTMM (6.1 g, 22 mmol) in acetonitrile (100 mL) was added to a solution of HCl·H-Pro-OBzl (4.8, 20 mmol) and triethylamine (3.3 mL, 24 mmol) in acetonitrile

(50 mL). The mixture was stirred at room temperature for 20 hours. Acetonitrile was removed by rotary evaporator and ethyl acetate (100 mL) was added to a mixture. The organic phase was washed with 10 wt% of citric acid aq. (50 mL × 2), saturated $NaHCO_3$ aq. (50 mL × 2) and saturated NaCl aq. (50 mL × 2), then dried with magnesium sulfate. After filtration, the filtrate was evaporated to remove solvent and dried under vacuum. Boc-Arg(NO_2)-Pro-OBzl was obtained as a colorless oily compound; yield 9.7 g (96 %). Boc-Arg(NO_2)-Pro-OBzl (9.7 g, 19.2 mmol) was incubated in mixture of TFA and dichloromethane (20 mL, 1:1 volume ratio) at room temperature for 1 hour. The solvent was removed by rotary evaporator. The compound was precipitated in diethyl ether and dried under vacuum. TFA·H-Arg(NO_2)-Pro-OBzl was obtained as a colorless oily compound; yield, 7.7 g (77 %). To a solution of TFA·H-Arg(NO_2)-Pro-OBzl (7.7 g, 14.8 mmol) and triethylamine (2.5 mL, 17.8 mmol) in acetonitrile (50 mL), a mixture of Boc-Pro-OH (3.2 g, 14.8 mmol) and DMTMM (4.5 g, 16.3 mmol) in acetonitrile (50 ml) was added and stirred at room temperature for 20 hours. Acetonitrile was removed by rotary evaporator and ethyl acetate (100 mL) was added to a mixture. The organic phase was washed with the aqueous solutions mentioned above, then dried with magnesium sulfate. After filtration, the filtrate was evaporated to remove solvent and dried under vacuum. Boc-Pro-Arg(NO_2)-Pro-OBzl was obtained as a pale-yellow oily compound; yield 7.3 g (82 %).

Boc-Pro-Arg(NO_2)-Pro-OBzl (7.3 g, 12.1 mmol) was incubated in mixture of TFA and dichloromethane (20 mL, 1:1 volume ratio) at room temperature for 1 hour. The solvent was removed by rotary evaporator. The compound was precipitated in diethyl ether and dried under vacuum. TFA·H-Pro-Arg(NO_2)-Pro-OBzl was obtained as a pale-yellow oily compound; yield, 6.4 g (85 %). To a solution of TFA·H-Pro-Arg(NO_2)-Pro-OBzl (6.4 g, 10.3 mmol) and triethylamine (1.7 mL, 12.4 mmol) in acetonitrile (50 mL), a mixture of Tos-Gly-OH (2.4 g, 10.3 mmol) and DMTMM (3.1 g, 11.3 mmol) in acetonitrile (50 ml) was added and stirred at room temperature for 20 hours. Acetonitrile was removed by rotary evaporator and ethyl acetate (100 mL) was added to a mixture. The organic phase was washed with the aqueous solutions and then dried with magnesium sulfate. After filtration, the filtrate was evaporated to remove solvent and dried under vacuum. Tos-Gly-Pro-Arg(NO_2)-Pro-OBzl was obtained as a pale-yellow amorphous compound; yield 7.0 g (95 %).

To a solution of Tos-Gly-Pro-Arg(NO_2)-Pro-OBzl (7.0 g, 9.8 mmol) in methanol (50 mL), 1 N NaOH aq. (12.0 mL) was slowly added and stirred at room temperature for 2 hours. Methanol was removed by rotary evaporator and water (50 mL) was added to the mixture. The water phase was washed with diethyl ether (20 mL × 2) and adjusted to pH 2 by 1 N HCl aq. The compound was extract with ethyl acetate (20 mL× 3), and the organic phase was washed with 10 wt% of citric acid aq. (20 mL × 2) and saturated NaCl aq. (20 mL × 2). The organic phase was dried with magnesium sulfate. After filtration, the filtrate was evaporated to remove solvent and dried under vacuum. Tos-Gly-Pro-Arg(NO_2)-Pro-OH was obtained as a colorless amorphous compound; yield 5.9 g (96%). To a solution of Tos-Gly-Pro-Arg(NO_2)-Pro-OH (1.2 g, 1.9 mmol) and DMTMM (640 mg, 2.3 mmol) in acetonitrile (50 mL), NH_2-PEG$_4$-COOtBu (710 mg, 2.2 mmol, Broadpharm, San Diego, CA, USA) and triethylamine (500 μL, 3.6 mmol) were added and the mixture was stirred at room temperature for 20 hours. After removing acetonitrile by rotary evaporator, ethyl acetate (50 mL) was added, and the organic phase was washed with 10 wt% citric acid aq. (20 mL × 2), saturated $NaHCO_3$ aq. (20 mL × 2), and saturated NaCl (20 mL × 2). After drying organic phase with $MgSO_4$, filtrate was evaporated by rotary evaporator to remove ethyl acetate. Tos-Gly-Pro-Arg(NO_2)-Pro-PEG$_4$-COOtBu was obtained as a pale-yellow oil; yield 1.2 g (68%). Tos-Gly-Pro-Arg(NO_2)-Pro-PEG$_4$-COOtBu (1.2 g, 1.3 mmol) and TFA (1.0 mL), 13 mmol) were dissolved in dichloromethane (20 mL), and then the mixture was stirred at room temperature for 1 hour. The compound was obtained by removing dichloromethane and TFA by rotary evaporator, followed by precipitating in diethyl ether. Tos-Gly-Pro-Arg(NO_2)-Pro-PEG$_4$-COOH was obtained as a pale-yellow oil after drying *in vacuum*; yield 0.8 g (75%). The chemical structure of Tos-Gly-Pro-Arg(NO_2)-Pro-PEG$_4$-COOH was confirmed by ^{1}H NMR in $CDCl_3$ (**Figure S1**).

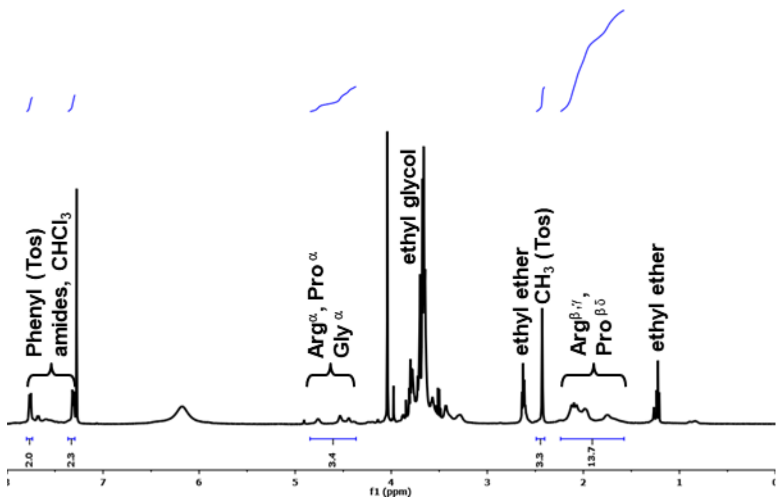

Figure S1. ^{1}H NMR spectrum of Tos-Gly-Pro-Arg(NO_2)-Pro-PEG_4-COOH in $CDCl_3$.

2) Synthesis of HCl·H-Ala-Ala-Pro-Phe-OBzl. A solution of Boc-Ala-OH (3.8 g, 20 mmol) and DMTMM (6.1 g, 22 mmol) in acetonitrile (100 mL) was added to a solution of TosOH H-Ala-OBzl (7.0 g, 20 mmol) and triethylamine (3.3 mL, 24 mmol) in acetonitrile (50 mL). The mixture was stirred at room temperature for 20 hours. Acetonitrile was removed by rotary evaporator and ethyl acetate (100 mL) was added to a mixture. The organic phase was washed with 10 wt% of citric acid aq. (50 mL × 2), saturated $NaHCO_3$ aq. (50 mL × 2) and saturated NaCl aq. (50 mL × 2), then dried with magnesium sulfate. After filtration, the filtrate was evaporated to remove solvent and dried under vacuum. Boc-Ala-Ala-OBzl was obtained as a white powder compound; yield 6.9 g (98 %). To a solution of Boc-Ala-Ala-OBzl (6.9 g, 19.6 mmol) in methanol (50 mL), 1 N NaOH aq. (2.2 mL) was added and stirred at room temperature for 2 hours. Methanol was removed by rotary evaporator and water (50 mL) was added to the mixture. The water phase was washed with diethyl ether (30 mL × 2) and adjusted to pH 2 by 1 N HCl aq. in the ice bath. The compound was extract with ethyl acetate (30 mL× 3), and the organic phase was washed with 10 wt% of citric acid aq. (30 mL × 2) and saturated NaCl aq. (30 mL × 2). The organic phase was dried with magnesium sulfate. Boc-Ala-Ala-OH was isolated by precipitation in mixture of ethyl acetate and diethyl ether (1:1 volume ratio); yield 4.2 g (85 %). A solution of Boc-Pro-OH (4.3 g, 20 mmol) and DMTMM (6.1 g, 22 mmol) in acetonitrile (100 mL) was added to a solution of TosOH·H-Phe-OBzl (8.6 g, 20 mmol) and triethylamine (3.3 mL, 24 mmol) in acetonitrile (50 mL). The mixture was stirred at room

temperature for 20 hours. Acetonitrile was removed by rotary evaporator and ethyl acetate (100 mL) was added to a mixture. The organic phase was washed with 10 wt% of citric acid aq. (50 mL × 2), saturated $NaHCO_3$ aq. (50 mL × 2) and saturated NaCl aq. (50 mL × 2), then dried with magnesium sulfate. After filtration, the filtrate was evaporated to remove solvent and dried under vacuum. Boc-Pro-Phe-OBzl was obtained as a pale yellow oily compound; yield 8.8 g (97 %). Boc-Pro-Phe-OBzl (8.8 g, 19.4 mmol) was incubated in 4M HCl in 1,4-dioxane (50 mL) at room temperature for 1 hour. The solvent was removed by rotary evaporator. The compound was precipitated in diethyl ether and dried under vacuum. HCl•H-Pro-Phe-OBzl was obtained as a white solid compound; yield 6.9 g (92 %). Boc-Ala-Ala-OH (3.2 g, 12 mmol) and DMTMM (3.9 g, 14 mmol) was added to acetonitrile (100 mL), and the solution was stirred at room temperature for 1 h. After added HCl·H-Pro-Phe-OBzl (4.2 g, 10.8 mmol) and triethylamine (2.1 mL, 15.0 mmol), the mixture was stirred at room temperature for 20 h. Acetonitrile was removed by rotary evaporator and ethyl acetate (100 mL) was added. The organic phase was washed with deionized water (30 mL × 2), 10 wt% citric acid aq. (30 mL × 2), saturated $NaHCO_3$ aq. (30 mL × 2), and saturated NaCl (30 mL × 2), and then dried with $MgSO_4$. The filtrate was evaporated to remove ethyl acetate, and the compound was dried in vacuum and obtained as pale yellow oil; yield 5.9 g (92 %). 4 M HCl in 1,4-dioxane (15 mL) was added to Boc-Ala-Ala-Pro-Phe-OBzl (3.6 g, 6.0 mmol) in a round bottom flask, and the mixture was stirred at room temperature for 1 h. After removing 1,4-dioxne, the compound was precipitated in diethyl ether. HCl·H-Ala-Ala-Pro-Phe-OBzl was dried under vacuum and obtained as pale yellow amorphous; yield 3.1 g (98 %). The chemical structure of HCl·H-Ala-Ala-Pro-Phe-OBzl was confirmed by ^{1}H NMR in $CDCl_3$ (**Figure S2**).

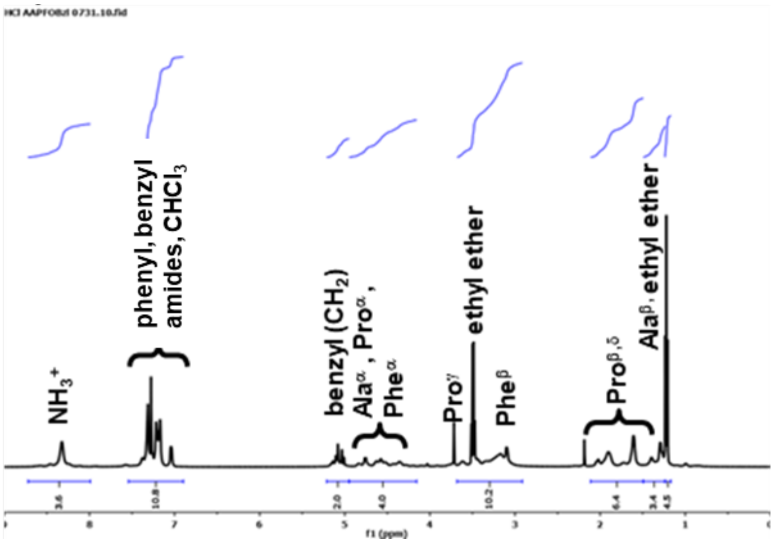

Figure S2. ^{1}H NMR spectrum of HCl·H-Ala-Ala-Pro-Phe-OBzl in $CDCl_3$.

3) Synthesis of HCl H-Ala-Ala-OEGMA. Boc-Ala-Ala-OEGMA was synthesized by esterification of Boc-Ala-Ala-OH with poly(ethylene glycol) methacrylate. To a solution of Boc-Ala-Ala-OH (1.0 g, 4.0 mmol) and NHS (580 μg, 5.0 mmol) in acetonitrile in ice bath, EDC·HCl (960 μg, 5.0 mmol) was added and stirred for 1 h. Poly(ethylene glycol) methacrylate (average M_n 360, 1.8 g, 5.0 mmol) and DMAP (730 μg, 6.0 mmol) were added to the solution, and the mixture was stirred in a refrigerator for 20 h. Acetonitrile was removed by rotary evaporator and dichloromethane (50 mL) to the mixture. The organic phase was washed with deionized water (20 mL × 2), 0.05 N NaOH aq. (20 mL × 2), 0.05 N HCl aq. (20 mL × 2), and saturated NaCl aq. (20 mL), and then the organic phase was dried with magnesium sulfate. After filtration, the filtrate was evaporated to remove solvent and dried under vacuum. Boc-Ala-Ala-OEGMA was obtained was as a colorless oily compound; yield 2.1 g (87 %). Boc-Ala-Ala-OEGMA (2.1 g, 3.5 mmol) was incubated in 4M HCl in 1,4-dioxane (8 mL) at room temperature for 1 h. The solvent was removed by rotary evaporator. The compound was precipitated in diethyl ether and dried under vacuum. HCl·H-Ala-Ala-OEGMA was obtained as a pale yellow oily compound; yield, 1.8 g (95 %). The chemical structure of the HCl·H-Ala-Ala-OEGMA was determined by ^{1}H NMR spectrum in DMSO-d_6 (**Figure S3**).

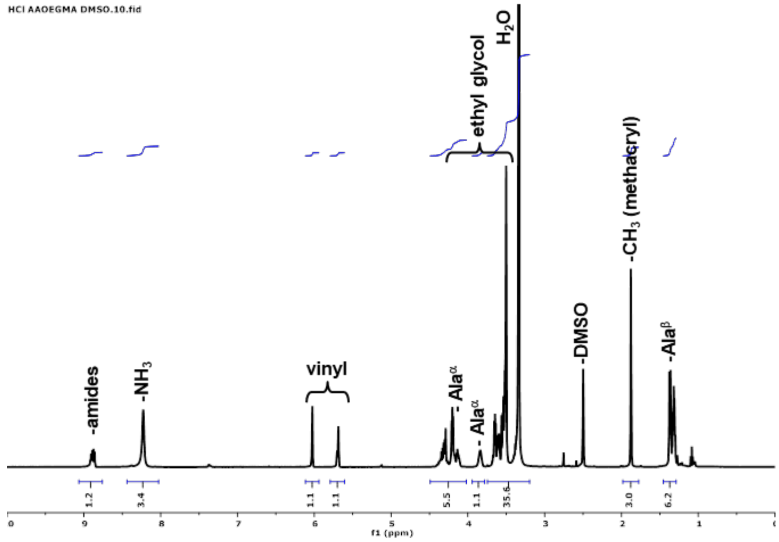

Figure S3. ^{1}H NMR spectrum of HCl·H-Ala-Ala-OEGMA in DMSO-d_6.

4) Synthesis of Tos-Gly-Pro-Arg-Pro-PEG$_4$-Ala-Ala-Pro-Phe-Ala-Ala-OEGMA. A solution of Tos-Gly-Pro-Arg(NO_2)-Pro-PEG$_4$-OH (800 mg, 0.98 mmol) and DMTMM (330 mg, 1.2 mmol) in acetonitrile (20 mL) was added to a solution of HCl·H-Ala-Ala-Pro-Phe-OBzl (530 mg, 1.0 mmol) and triethylamine (200 μL, 1.4 mmol) in the mixture of acetonitrile (50 mL) and *N,N*-dimethylformamide (5 mL). The mixture was stirred at room temperature for 20 hours. Acetonitrile was removed by rotary evaporator and ethyl acetate (50 mL) was added to a mixture. The organic phase was washed with 10 wt% of citric acid aq. (20 mL × 2), saturated $NaHCO_3$ aq. (20 mL × 2) and saturated NaCl aq. (20 mL × 2), then dried with magnesium sulfate. After filtration, the filtrate was evaporated to remove solvent and dried under vacuum. To a solution the crude compound in methanol (20 mL), ammonium formate (320 mg, 5.0 mmol) and Pd/C (10 wt%, 150 mg) were added. The mixture was stirred at room temperature for 20 h. After filtration, Tos-Gly-Pro-Arg-Pro-PEG$_4$-Ala-Ala-Pro-Phe-OH was purified through dialysis (500 - 1,000 Da MWCO) against the mixture of methanol and deionized water (1:1 volume ratio) and deionized water at room temperature for 24 hours and then lyophilized. To a solution of Tos-Gly-Pro-Arg-Pro-PEG$_4$-Ala-Ala-Pro-Phe-OH (740 mg, 0.61 mmol) and DMTMM (220 mg, 0.8 mmol) in acetonitrile (10 mL), HCl·H-Ala-Ala-OEGMA (530 mg, 1.0 mmol) and triethylamine (140 μL, 1.0 mmol) were added and the mixture was stirred in a refrigerator for 20 hours. Tos-Gly-Pro-Arg-Pro-PEG$_4$-Ala-Ala-Pro-Phe-Ala-Ala-OEGMA was purified through dialysis (1 kDa

MWCO) against the mixture of methanol and deionized water (1:1 volume ratio) and deionized water at room temperature for 24 hours and then lyophilized; yield, 750 mg (72%). The chemical structure of the Tos-Gly-Pro-Arg-Pro-PEG_4-Ala-Ala-Pro-Phe-Ala-Ala-OEGMA was determined by ^{1}H NMR spectrum in D_2O(**Figure S4**).

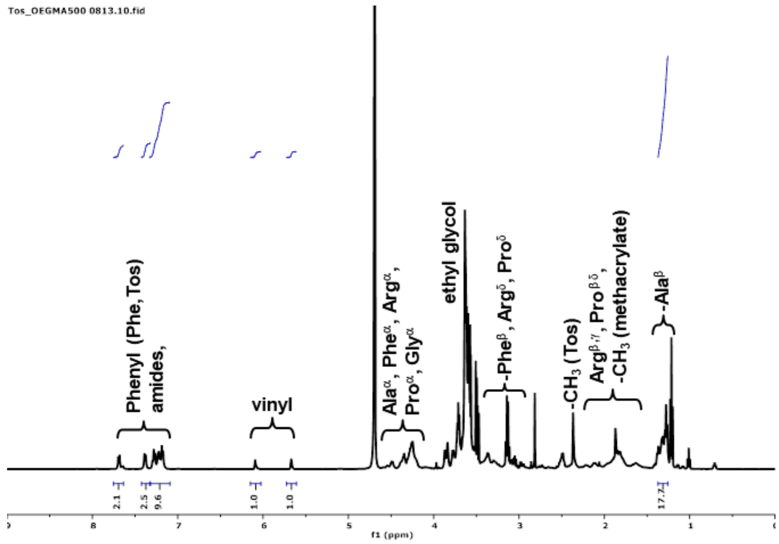

Figure S4. ^{1}H NMR spectrum of macromonomer containing TR inhibitor and CT cleavable peptide moieties (Tos-Gly-Pro-Arg-Pro-PEG_4-Ala-Ala-Pro-Phe-Ala-Ala-OEGMA) in D_2O.

Synthesis of macromonomer containing CT inhibitor and TR cleavable peptide moieties (IB-Ala-Ala-Pro-Phe-Sar-PEG_4-Gly-Pro-Arg-Gly-OEGMA). A macromonomer containing a trypsin inhibitor and a chymotrypsin-cleavable peptide moieties was synthesized using the procedure shown below (**Scheme S2**).

Scheme S2. Synthetic scheme for macromonomer containing CT inhibitor and TR cleavable peptide moieties (IB-Ala-Ala-Pro-Phe-Sar-PEG$_4$-Gly-Pro-Arg-Gly-Gly-OEGMA).

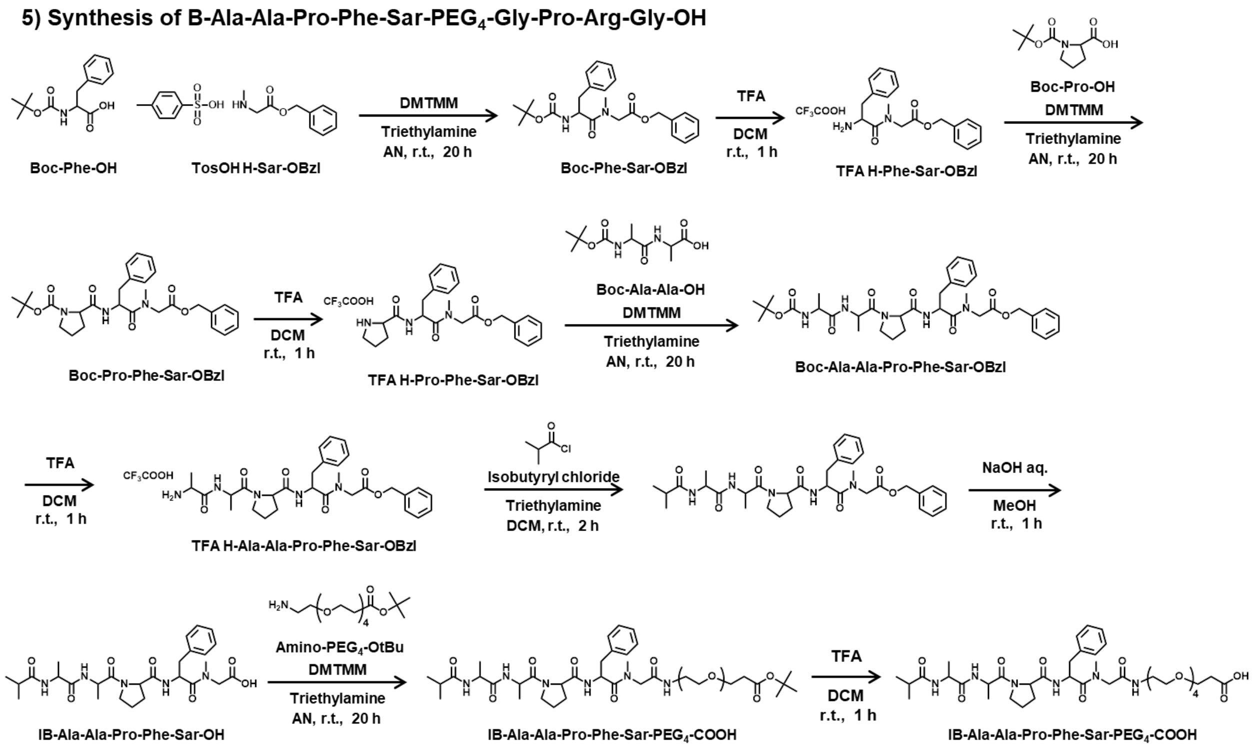

6) Synthesis of TFA H-Gly-Pro-Arg(NO_2)-Gly-OBzl

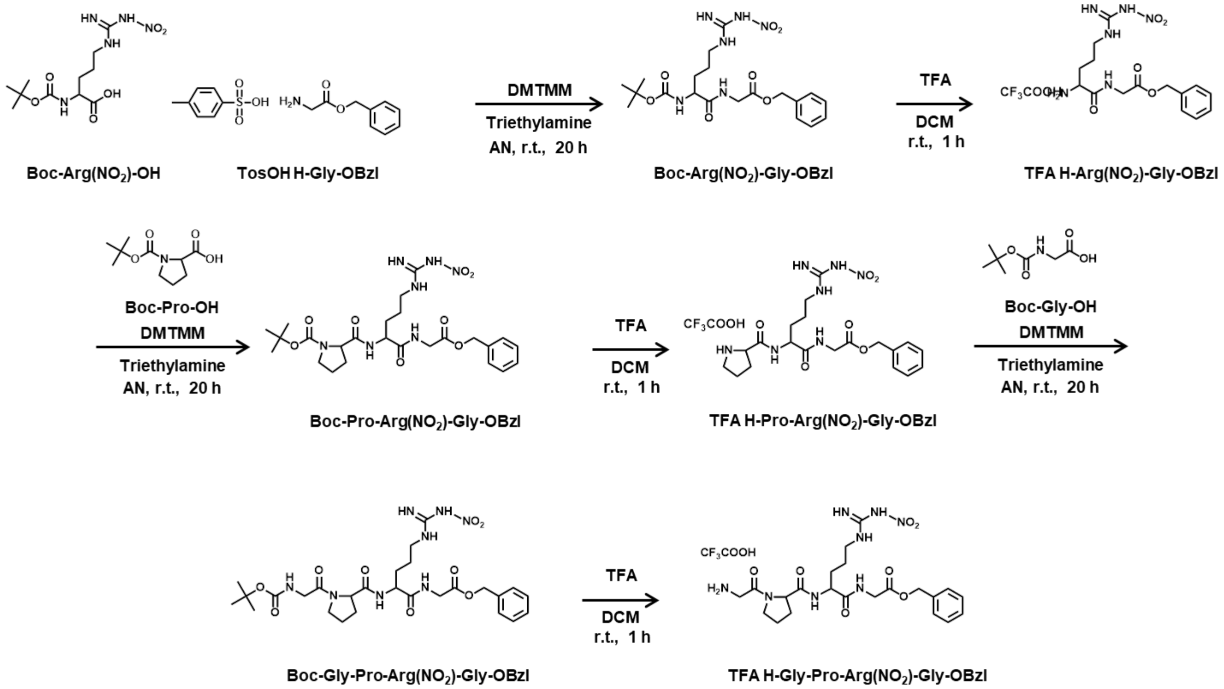

7) Synthesis of IB-Ala-Ala-Pro-Phe-Sar-PEG4-Gly-Pro-Arg-Gly-OH

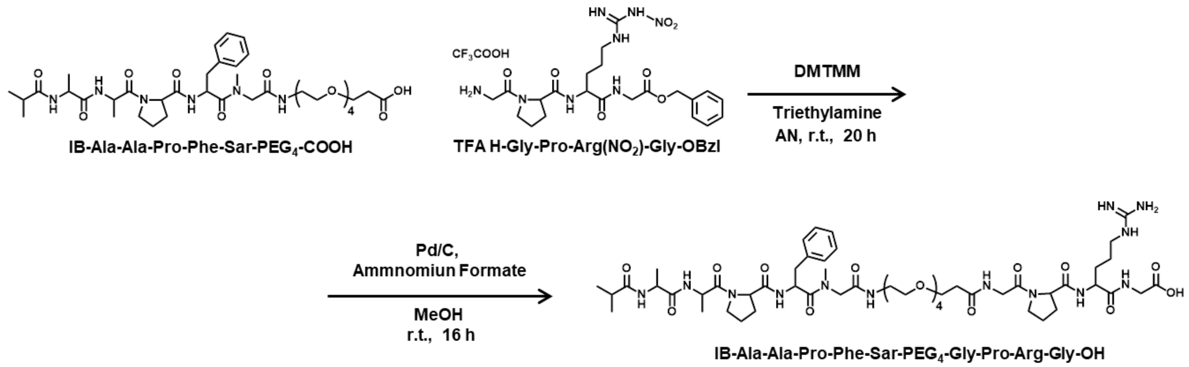

8) Synthesis of macro-monomer IB-Ala-Ala-Pro-Phe-Sar-PEG4-Gly-Pro-Arg-Gly-Gly-OEGMA

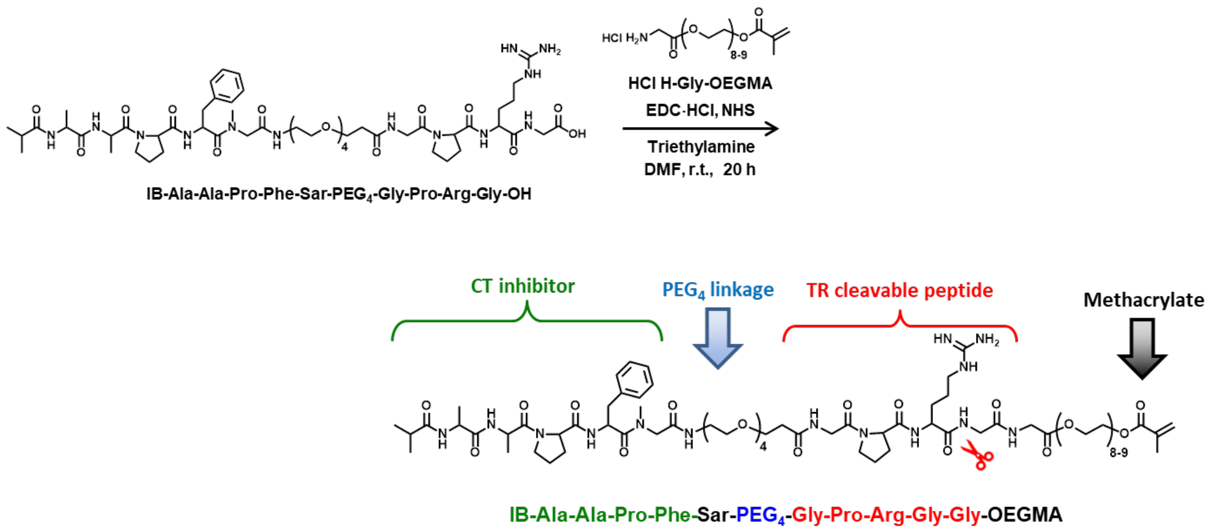

5) Synthesis of IB-Ala-Ala-Pro-Phe-Sar-PEG$_4$-COOH. A solution of Boc-Phe-OH (5.3 g, 20 mmol) and DMTMM (6.1 g, 22 mmol) in acetonitrile (100 mL) was added to a solution of TosOH·H-Sar-OBzl (7.0 g, 20 mmol) and triethylamine (3.3 mL, 24 mmol) in acetonitrile (50 mL). The mixture was stirred at room temperature for 20 hours. Acetonitrile was removed by rotary evaporator and ethyl acetate (100 mL) was added to a mixture. The organic phase was washed with 10 wt% of citric acid aq. (50 mL × 2), saturated $NaHCO_3$ aq. (50 mL × 2) and saturated NaCl aq. (50 mL × 2), then dried with magnesium sulfate. After filtration, the filtrate was evaporated to remove solvent and dried under vacuum. Boc-Phe-Sar-OBzl was obtained as a colorless oily compound; yield 7.3 g (86 %). Boc-Phe-Sar-OBzl (7.3 g, 17.2 mmol) was incubated in mixture of TFA and dichloromethane (20 mL, 1:1 volume ratio) at room temperature for 1 hour. The solvent was removed by rotary evaporator. The compound was precipitated in diethyl ether and dried under vacuum. TFA·H-Phe-Sar-OBzl was obtained as a colorless oily compound; yield, 6.0 g (79 %). To a solution of TFA·H-Phe-Sar-OBzl (6.0 g, 13.6 mmol) and triethylamine (2.3 mL, 16.3 mmol) in acetonitrile (50 mL), a mixture of Boc-Pro-OH (2.9 g, 13.6 mmol) and DMTMM (4.2 g, 15.0 mmol) in acetonitrile (50 ml) was added and stirred at room temperature for 20 hours. Acetonitrile was removed by rotary evaporator and ethyl acetate (100 mL) was added to a mixture. The organic phase was washed with the aqueous solutions mentioned above, then dried with magnesium sulfate. After filtration, the filtrate was evaporated to remove solvent and dried under vacuum. Boc-Pro-Phe-Sar-OBzl was obtained as

a pale yellow oily compound; yield 6.9 g (96 %). Boc-Pro-Phe-Sar-OBzl (6.9 g, 13.1 mmol) was incubated in mixture of TFA and dichloromethane (20 mL, 1:1 volume ratio) at room temperature for 1 hour. The solvent was removed by rotary evaporator. The compound was precipitated in diethyl ether and dried under vacuum. TFA·H-Pro-Phe-Sar-OBzl was obtained as a colorless oily compound; yield, 6.3 g (89 %). To a solution of TFA·H-Pro-Phe-Sar-OBzl (6.3 g, 11.7 mmol) and triethylamine (2.0 mL, 14.0 mmol) in acetonitrile (50 mL), a mixture of Boc-Ala-Ala-OH (3.0 g, 11.7 mmol) and DMTMM (3.6 g, 12.9 mmol) in acetonitrile (50 ml) was added and stirred at room temperature for 20 hours. Acetonitrile was removed by rotary evaporator and ethyl acetate (100 mL) was added to a mixture. The organic phase was washed with the aqueous solutions mentioned above, then dried with magnesium sulfate. After filtration, the filtrate was evaporated to remove solvent and dried under vacuum. Boc-Ala-Ala-Pro-Phe-Sar-OBzl was obtained as a white powder compound; yield 6.3 g (81 %). Boc-Ala-Ala-Pro-Phe-Sar-OBzl (6.3 g, 9.5 mmol) was incubated in mixture of TFA and dichloromethane (20 mL, 1:1 volume ratio) at room temperature for 1 hour. The solvent was removed by rotary evaporator. The compound was precipitated in diethyl ether and dried under vacuum. TFA·H-Ala-Ala-Pro-Phe-Sar-OBzl was obtained as a pale yellow oily compound; yield, 5.0 g (77 %). To a solution of TFA·H-Ala-Ala-Pro-Phe-Sar-OBzl (5.0 g, 7.3 mmol) and triethylamine (1.2 mL, 8.8 mmol) in dichloromethane (50 mL), a solution of isobutyryl chloride (840 μL, 8.0 mmol) in dichloromethane (50 ml) was slowly added in the ice bath and stirred at room temperature for 20 hours. The organic phase was washed with the aqueous solutions mentioned above and then dried with magnesium sulfate. After filtration, the filtrate was evaporated to remove solvent and dried under vacuum. IB-Ala-Ala-Pro-Phe-Sar-OBzl was obtained as a white powder compound; yield 4.6 g (97 %). To a solution of IB-Ala-Ala-Pro-Phe-Sar-OBzl (4.6 g, 7.1 mmol) in methanol (50 mL), 1 N NaOH aq. (7.5 mL) was slowly added and stirred at room temperature for 2 hours. Methanol was removed by rotary evaporator and water (30 mL) was added to the mixture. The water phase was washed with diethyl ether (30 mL × 2) and adjusted to pH 2 by 1 N HCl aq. The compound was extract with ethyl acetate (30 mL× 3), and the organic phase was washed with 10 wt% of citric acid aq. (20 mL × 2) and saturated NaCl aq. (20 mL × 2). The organic phase was dried with magnesium sulfate. IB-Ala-Ala-Pro-Phe-Sar-OH was isolated by precipitation in diethyl ether; yield 3.0 g (74 %). A solution of IB-Ala-Ala-Pro-Phe-Sar-OH (1.3 g, 2.3 mmol) and DMTMM (830 mg, 3.0 mmol) in DMF (10 mL) was added to the solution of

AminoPEG4-OtBu (1.0 g, 3.1 mmol) and triethylamine (500 μL, 3.6 mmol) in DMF (5 mL) and the mixture was stirred at room temperature for 20 hours. DMF was removed by rotary evaporator and ethyl acetate (50 mL) was added to a mixture. The organic phase was washed with the aqueous solutions mentioned above, then dried with magnesium sulfate. After filtration, the filtrate was evaporated to remove solvent and dried under vacuum. IB-Ala-Ala-Pro-Phe-Sar-PEG4-OtBu was obtained as a pale yellow amorphous; yield 1.7 g (87 %). IB-Ala-Ala-Pro-Phe-Sar-PEG4-OtBu (1.7 g, 2.0 mmol) and TFA (1.0 mL) were dissolved in dichloromethane (20 mL), and then the mixture was stirred at room temperature for 1 hour. The compound was obtained by removing dichloromethane and TFA by rotary evaporator, followed by precipitating in diethyl ether. A crude IB-Ala-Ala-Pro-Phe-Sar-PEG4-COOH was obtained by precipitation in acetone; yield g 1.5 g (95%). The chemical structure of the IB-Ala-Ala-Pro-Phe-Sar-PEG4-COOH was determined by ^{1}H NMR spectrum in DMSO-d_6 (**Figure S5**).

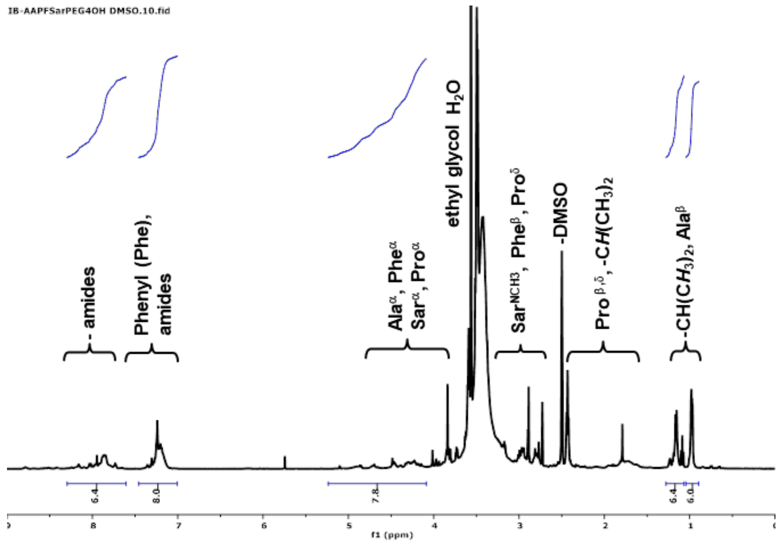

Figure S5. ^{1}H NMR spectrum of IB-Ala-Ala-Pro-Phe-Sar-PEG$_4$-COOH in DMSO-d_6.

6) Synthesis of TFA·H-Gly-Pro-Arg(NO$_2$)-Gly-OBzl. A solution of Boc-Arg(NO$_2$)-OH (6.4 g, 20 mmol) and DMTMM (6.1 g, 22 mmol) in acetonitrile (100 mL) was added to a solution of TosOH·H-Gly-OBzl (6.7 g, 20 mmol) and triethylamine (3.3 mL, 24 mmol) in acetonitrile (50 mL). The mixture was stirred at room temperature for 20 hours. Acetonitrile was removed by rotary evaporator and ethyl acetate (100 mL) was added to a mixture. The organic phase was washed with 10 wt% of citric acid aq. (50 mL × 2), saturated $NaHCO_3$ aq. (50 mL × 2) and

saturated NaCl aq. (50 mL × 2), then dried with magnesium sulfate. After filtration, the filtrate was evaporated to remove solvent and dried under vacuum. Boc-Arg(NO_2)-Gly-OBzl was obtained as a colorless oily compound; yield 7.7 g (83 %). Boc-Arg(NO_2)-Gly-OBzl (7.7 g, 16.6 mmol) was incubated in mixture of TFA and dichloromethane (20 mL, 1:1 volume ratio) at room temperature for 1 hour. The solvent was removed by rotary evaporator. The compound was precipitated in diethyl ether and dried under vacuum. TFA·H-Arg(NO_2)-Gly-OBzl was obtained as a colorless oily compound; yield, 6.5 g (81 %). To a solution of TFA·H-Arg(NO_2)-Gly-OBzl (6.5 g, 13.6 mmol) and triethylamine (2.3 mL, 16.3 mmol) in acetonitrile (50 mL), a mixture of Boc-Pro-OH (2.9 g, 13.6 mmol) and DMTMM (4.2 g, 15.0 mmol) in acetonitrile (50 ml) was added and stirred at room temperature for 20 hours. Acetonitrile was removed by rotary evaporator and ethyl acetate (100 mL) was added to a mixture. The organic phase was washed with the aqueous solutions mentioned above, then dried with magnesium sulfate. After filtration, the filtrate was evaporated to remove solvent and dried under vacuum. Boc-Pro-Arg(NO_2)-Gly-OBzl was obtained as a pale-yellow oily compound; yield 7.0 g (92 %). Boc-Pro-Arg(NO_2)-Gly-OBzl (7.0 g, 12.5 mmol) was incubated in mixture of TFA and dichloromethane (20 mL, 1:1 volume ratio) at room temperature for 1 hour. The solvent was removed by rotary evaporator. The compound was precipitated in diethyl ether and dried under vacuum. TFA·H-Pro-Arg(NO_2)-Gly-OBzl was obtained as a pale-yellow oily compound; yield, 5.5 g (77 %). To a solution of TFA·H-Pro-Arg(NO_2)-Gly-OBzl (5.5 g, 9.6 mmol) and triethylamine (1.7 mL, 12.0 mmol) in acetonitrile (50 mL), a mixture of Boc-Gly-OH (1.8 g, 10.0 mmol) and DMTMM (3.0 g, 11.0 mmol) in acetonitrile (50 ml) was added and stirred at room temperature for 20 hours. Acetonitrile was removed by rotary evaporator and ethyl acetate (100 mL) was added to a mixture. The organic phase was washed with the aqueous solutions mentioned above, then dried with magnesium sulfate. After filtration, the filtrate was evaporated to remove solvent and dried under vacuum. Boc-Gly-Pro-Arg(NO_2)-Gly-OBzl was obtained as a pale-yellow amorphous compound; yield 5.9 g (99 %). Boc-Gly-Pro-Arg(NO_2)-Gly-OBzl (5.9 g, 9.5 mmol) was incubated in mixture of TFA and dichloromethane (20 mL, 1:1 volume ratio) at room temperature for 1 hour. The solvent was removed by rotary evaporator. The compound was precipitated in diethyl ether and dried under vacuum. TFA· H-Gly-Pro-Arg(NO_2)-Gly-OBzl was obtained as a pale yellow amorphous compound; yield, 4.1 g (68 %). The chemical structure of

the TFA· H-Gly-Pro-Arg(NO_2)-Gly-OBzl was determined by ^{1}H NMR spectrum in DMSO-d_6 (**Figure S6**).

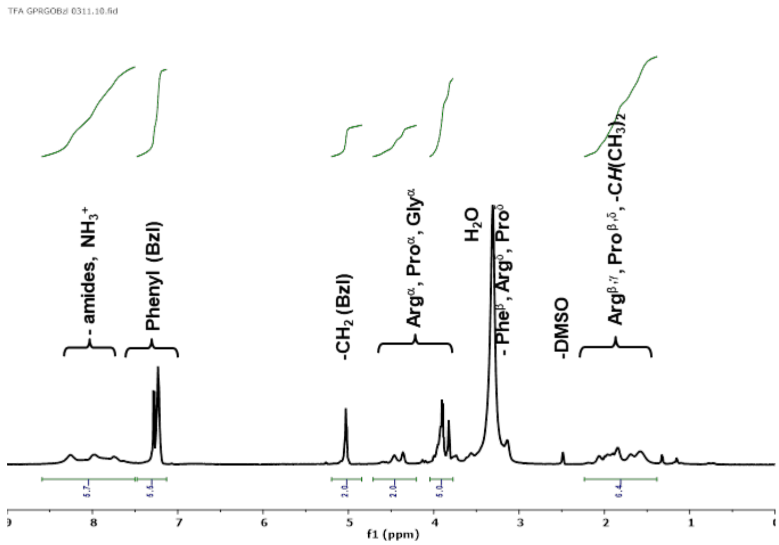

Figure S6. ^{1}H NMR spectrum of TFA·H-Gly-Pro-Arg(NO_2)-Gly-OBzl in DMSO-d_6.

7) Synthesis of IB-Ala-Ala-Pro-Phe-Sar-PEG$_4$-Gly-Pro-Arg-Gly-OH. A solution of IB-Ala-Ala-Pro-Phe-Sar-PEG$_4$-COOH (800 mg, 1.0 mmol) and DMTMM (330 mg, 1.2 mmol) in DMF (5 mL) was added to a solution of TFA· H-Gly-Pro-Arg(NO_2)-Gly-OBzl (700 mg, 1.1 mmol) and triethylamine (210 μL, 1.5 mmol) in acetonitrile (10 mL). The mixture was stirred at room temperature for 20 hours. Acetonitrile was removed by rotary evaporator. To a solution the crude IB-Ala-Ala-Pro-Phe-Sar-PEG$_4$-Gly-Pro-Arg(NO_2)-Gly-OBzl in methanol (25 mL), ammonium formate (320 mg, 5.0 mmol) and Pd/C (10 wt%, 150 mg) were added. The mixture was stirred at room temperature for 20 hours. After filtration, IB-Ala-Ala-Pro-Phe-Sar-PEG$_4$-Gly-Pro-Arg-Gly-OH was purified through dialysis (500 - 1,000 Da MWCO) against the mixture of methanol and deionized water (1:1 volume ratio) and deionized water at room temperature for 24 hours and then lyophilized. IB-Ala-Ala-Pro-Phe-Sar-PEG$_4$-Gly-Pro-Arg-Gly-OH was obtained as white amorphous compound; yield 920 mg (72 %). The chemical structure of the IB-Ala-Ala-Pro-Phe-Sar-PEG$_4$-Gly-Pro-Arg-Gly-OH was determined by ^{1}H NMR spectrum in DMSO-d_6 (**Figure S7**).

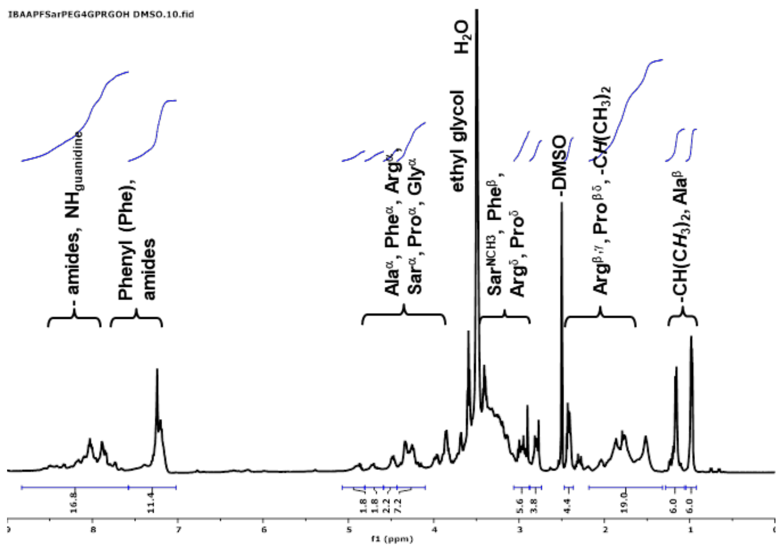

Figure S7. ^{1}H NMR spectrum of IB-Ala-Ala-Pro-Phe-Sar-PEG$_4$-Gly-Pro-Arg-Gly-OH in DMSO-d$_6$.

8) Synthesis of macromonomer containing TR inhibitor and CT cleavable peptide moieties (IB-Ala-Ala-Pro-Phe-Sar-PEG$_4$-Gly-Pro-Arg-Gly-Gly-OEGMA). A solution of IB-Ala-Ala-Pro-Phe-Sar-PEG$_4$-Gly-Pro-Arg-Gly-OH (450 mg, 0.35 mmol), *N*-hydroxylsuccinimide (58 mg, 0.5 mmol) and EDC·HCl (96 mg, 0.5 mmol) in DMF (5 mL) in ice bath, HCl·H-Gly-OEGMA (300 mg, 0.5 mmol) and triethylamine (85 μL, 0.6 mmol) were added and the mixture was stirred in a refrigerator for 20 hours.[1] IB-Ala-Ala-Pro-Phe-Sar-PEG$_4$-Gly-Pro-Arg-Gly-Gly-OEGMA was purified through dialysis (1 kDa MWCO) against the mixture of methanol and deionized water (1:1 volume ratio) and deionized water at room temperature for 24 hours and then lyophilized; yield, 390 mg (62%). The chemical structure of the IB-Ala-Ala-Pro-Phe-Sar-PEG$_4$-Gly-Pro-Arg-Gly-Gly-OEGMA was determined by ^{1}H NMR spectrum in DMSO-d$_6$ (**Figure S8**).

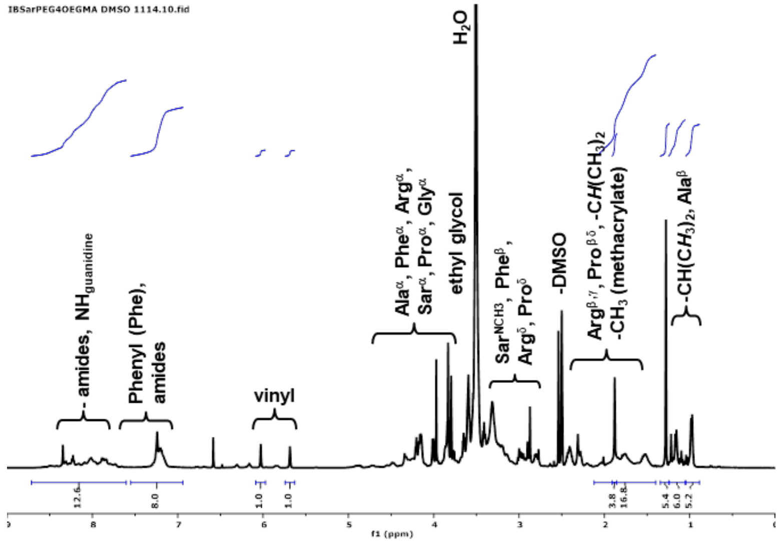

Figure S8. ^{1}H NMR spectrum of IB-Ala-Ala-Pro-Phe-Sar-PEG$_4$-Gly-Pro-Arg-Gly-Gly-OEGMA in DMSO-d$_6$.

Comparison of K_M of peptide substrates and K_i of model peptide inhibitors and macromonomers against native trypsin and chymotrypsin.

By observing the initial rate of enzyme activity in the presence of the peptide inhibitor or macromonomer, the K_i value of the inhibitor peptide and macromonomers can be determined (**Figures S9** for native trypsin,and **S1**4 for native chymotrypsin). The apparent K_M and V_{max} values are obtained by simultaneously mixing native trypsin or chymotrypsin, a peptide substrate, and a peptide inhibitor, and then monitoring the pNA produced by enzymatically catalyzed hydrolysis of the peptide substrate. The apparent K_M and V_{max} values were calculated using EnzFitter with Michaelis-Menten curve fitting of substrate against initial velocity plots (**Figure S10** and **S12** and **Table S1** and **S2** for native trypsin, and **Figure S15** and **S17** and **Table S3** and **S4** for native chymotrypsin). The plot of $K_{M\ ass}/V_{max\ app}$ versus $[I]_0$ or $[macromonomers]_0$ yields a straight line with a y-intercept of K_M/V_{max} and a slope of $K_M/(V_{max} \cdot K_i)$ (**Figures S11** and **S13** for native trypsin and **S16** and **S18** for native chymotrypsin, respsctivery). The inhibition constant (K_i) of the peptide inhibitor or macromonomer was calculated by simply dividing the y-intercept by the slope.

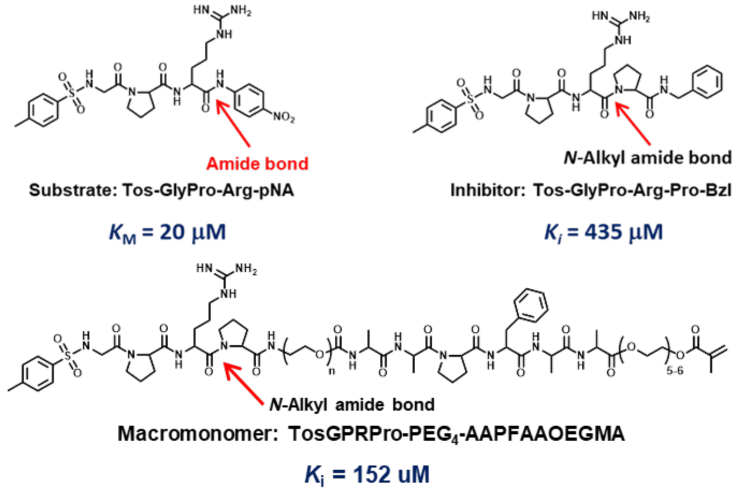

Figure S9. K_M value of the peptide substrate and K_i values of inhibitor peptide and macromonomer containing a CT-cleavable and a TR-inhibiting peptides (TosGPRPro-PEG$_4$-AAPFAA OEGMA) for native trypsin.

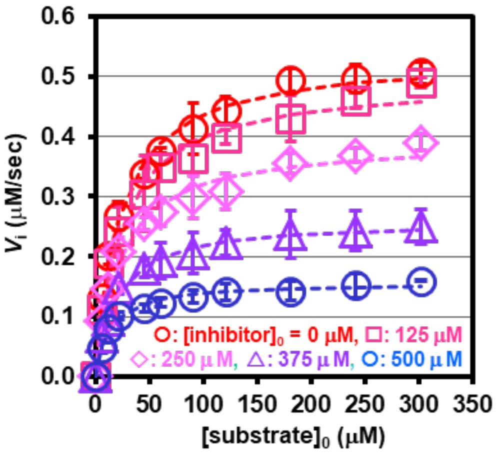

Figure S10. Michaelis-Menten Plots adding a peptide inhibitor to a native trypsin solution.

Table S1. Apparent K_M and V_{max} values for native trypsin incubated with Tos-GPR-pNA (substrate) and Tos-GPRP-NHBzl (inhibitor) at 37 °C in 100 mM Tris-HCl and 20 mM $CaCl_2$ buffer (pH 8.0)

	[inhibitor]$_0$ (μM)				
**Michaelis-Menten parameter**	**0**	**125**	**250**	**375**	**500**
K_M (μM)	22.2 ± 1.9	23.8 ± 2.8	22.1 ± 2.7	19.1 ± 1.3	13.2 ± 1.3
V_{max} (μM s^{-1})	0.53 ± 0.01	0.49 ± 0.01	0.39 ± 0.01	0.26 ± 0.01	0.16 ± 0.01
k_{cat} (s^{-1})	63.5± 1.3	58.8 ± 1.7	46.7 ± 1.3	30.9 ± 0.5	18.7 ± 0.4
k_{cat}/K_M (μM^{-1} sec^{-1})	2.87 ± 0.25	2.47 ± 0.30	2.11 ± 0.27	1.62 ± 0.12	1.41 ± 0.14
K_M /V_{max} (s)	41.5 ± 2.5	48.3 ± 4.1	56.3 ± 5.0	73.4 ± 3.7	84.3 ± 6.0

Buffer: 100 mM Tris-HCl and 20 mM $CaCl_2$ (pH 8.0) at 37 °C, $[TR]_0$ = 8.4 nM, $[TosGPRpNA]_0$ = 0 – 302 μ M and $[TosGPRPBzl]_0$ = 0 – 500 μM, Monitor Abs at 412 nm.

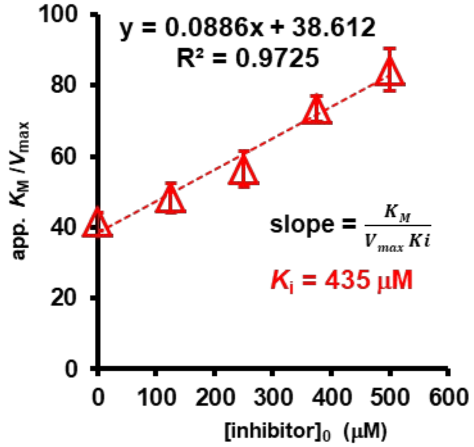

Figure S11. Secondary plot with calculated apparent K_M and V_{max} values were used to determine inhibition constants of the peptide inhibitor towards native trypsin.

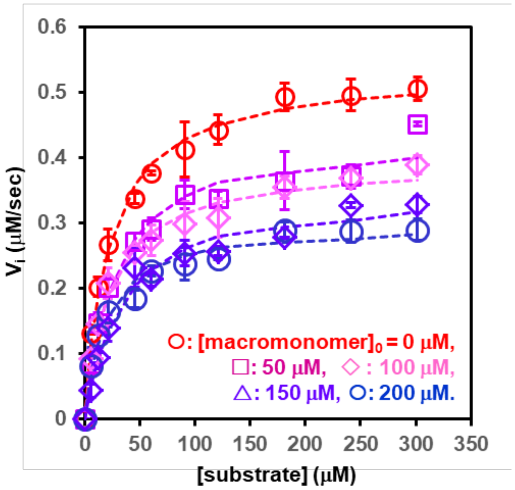

Figure S12. Michaelis-Menten Plots adding a the macromonomer containing a CT-cleavable and a TR-inhibiting peptides (TosGPRPro-PEG$_4$-AAPFAA OEGMA) to a native trypsin solution.

Table S2. Apparent K_M and V_{max} values for native trypsin incubated with Tos-GPR-pNA (substrate) and the macromonomer containing a CT-cleavable and a TR-inhibiting peptides (TosGPRPro-PEG$_4$-AAPFAA OEGMA) at 37 °C in 100 mM Tris-HCl and 20 mM $CaCl_2$ buffer (pH 8.0).

	**[macromonomer]$_0$ (μM)**				
**Michaelis-Menten parameter**	**0**	**50**	**100**	**150**	**200**
**K_M (μM)**	**22.2 ± 1.9**	**28.2 ± 4.5**	**18.8 ± 2.1**	**26.4 ± 3.2**	**35.5 ± 6.0**
**V_{max} (μM s^{-1})**	**0.53 ± 0.01**	**0.45 ± 0.02**	**0.24 ± 0.01**	**0.31 ± 0.01**	**0.36 ± 0.02**
**k_{cat} (s^{-1})**	**63.5± 1.3**	**53.4 ± 2.3**	**28.7 ± 0.7**	**36.5 ± 1.4**	**43.3 ± 2.1**
**k_{cat}/K_M (μM^{-1} sec^{-1})**	**2.87 ± 0.25**	**1.89 ± 0.31**	**1.53 ± 0.17**	**1.38 ± 0.18**	**1.22 ± 0.21**
**K_M/V_{max} (s)**	**41.5 ± 2.5**	**62.9 ± 7.3**	**77.9 ± 6.3**	**86.3 ± 7.8**	**97.7 ± 12.1**

Buffer: 100 mM Tris-HCl and 20 mM $CaCl_2$ (pH 8.0) at 37 °C, $[TR]_0$ = 8.4 nM, $[TosGPRpNA]_0$ = 0 – 241 μ M and $[macromonomer]_0$ = 0 – 200 μM, Monitor Abs at 412 nm.

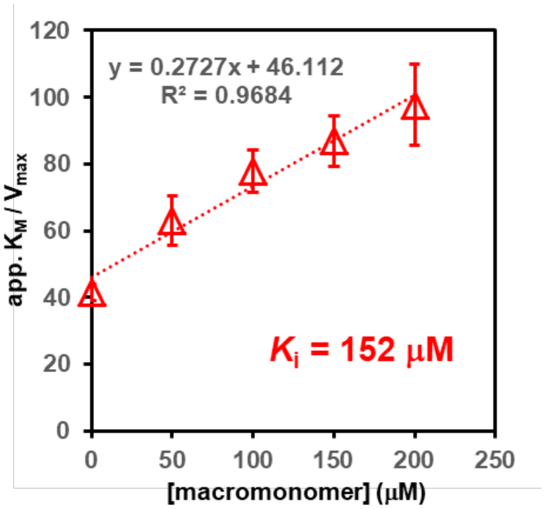

Figure S13. Secondary plot with calculated apparent K_M and V_{max} values were used to determine inhibition constants of the macromonomer containing a CT-cleavable and a TR-inhibiting peptides (TosGPRPro-PEG$_4$-AAPFAA OEGMA) towards native trypsin.

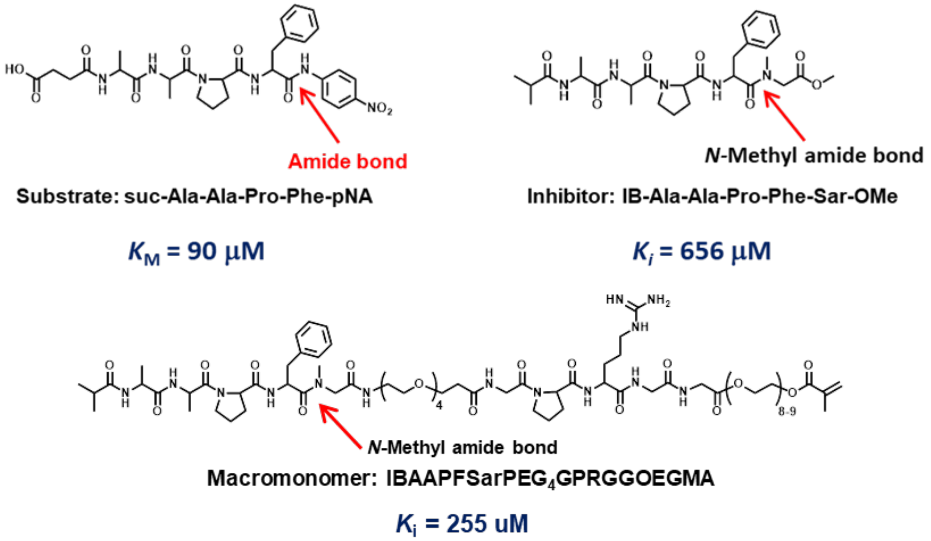

Figure S14. K_M value of peptide substrate and K_i values inhibitor peptide and macromonomer containing CT-cleavable and TR-inhibiting peptides (TosGPRPro-PEG$_4$-AAPFAA OEGMA) for native chymotrypsin.

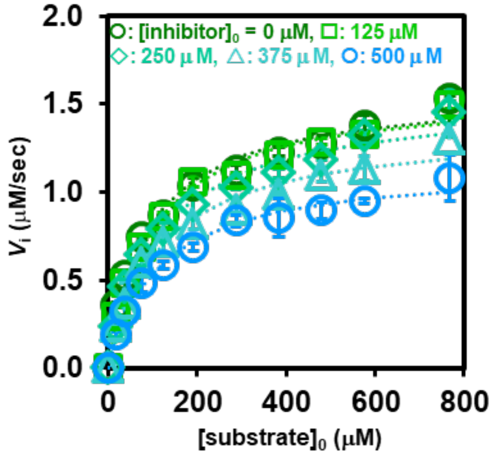

Figure S15. Michaelis-Menten Plots adding a peptide inhibitor to a native chymotrypsin solution.

Table S3. Apparent K_M and V_{max} values for native trypsin incubated with suc-AAPF-pNA (substrate) and IB-AAPFSar-OMe (inhibitor) at 37 °C in 100 mM sodium phosphate buffer (pH 8.0)

	[inhibitor]$_0$ (μM)				
**Michaelis-Menten parameter**	**0**	**125**	**250**	**375**	**500**
K_M (μM)	**90.1 ± 12.5**	**99.0 ± 10.4**	**116.7 ± 16.9**	**115.9 ± 16.6**	**114.8 ± 12.9**
V_{max} (μM s^{-1})	**1.56 ± 0.06**	**1.57 ± 0.05**	**1.54 ± 0.07**	**1.37 ± 0.06**	**1.15 ± 0.04**
k_{cat} (s^{-1})	**43.6 ± 1.5**	**39.3 ± 1.4**	**38.4 ± 1.6**	**34.2 ± 1.4**	**28.8 ± 1.0**
k_{cat}/K_M (μM^{-1} sec^{-1})	**0.48 ± 0.06**	**0.40 ± 0.05**	**0.33 ± 0.03**	**0.29 ± 0.03**	**0.25 ± 0.02**
K_M /V_{max} (s)	**57.4 ± 8.3**	**62.9 ± 6.8**	**75.9 ± 11.5**	**84.8 ± 12.7**	**99.6 ± 11.7**

Buffer: 100 mM sodium phosphate (pH 8.0) at 37 °C, [CT]$_0$ = 40 nM, [sucAAPFpNA]$_0$ = 0 – 768 μ M and [IBAAPFSarOMe]$_0$ = 0 – 500 μM, Monitor Abs at 412 nm.

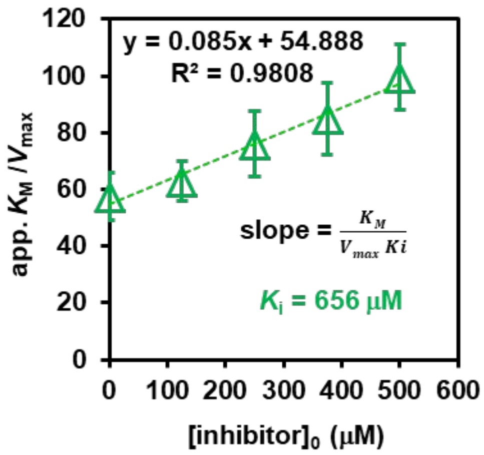

Figure S16. Secondary plot with calculated apparent K_M and V_{max} values were used to determine inhibition constants of the peptide inhibitor towards native chymotrypsin.

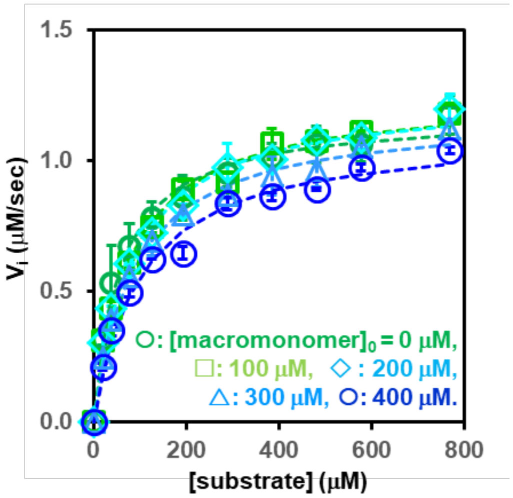

Figure S17. Michaelis-Menten Plots adding a macromonomer containing a TR cleavable and CT inhibiting peptides (IBAAPFSarPEG$_4$GPRGGOEGMA) to a native chymotrypsin solution.

Table S4. Apparent K_M and V_{max} values for native trypsin incubated with suc-AAPF-pNA (substrate) and macromonomer containing a TR cleavable and CT inhibiting peptides (IBAAPFSarPEG$_4$GPRGGOEGMA) at 37 °C in 100 mM sodium phosphate buffer (pH 8.0).

Michaelis-Menten parameter	[macromonomer]$_0$ (μM) 0	100	200	300	400
K_M (μM)	56.6 ± 6.0	77.5 ± 7.6	82.1 ± 8.4	86.1 ± 8.1	99.7 ± 12.6
V_{max} (μM s^{-1})	1.18 ± 0.03	1.25 ± 0.03	1.25 ± 0.03	1.18 ± 0.03	1.12 ± 0.04
k_{cat} (s^{-1})	29.5 ± 0.7	34.8 ± 0.8	34.9 ± 0.8	32.8 ±0.7	31.0 ± 1.0
k_{cat}/K_M (μM^{-1} sec^{-1})	0.52 ± 0.06	0.45 ± 0.05	0.43 ± 0.04	0.38 ± 0.73	0.31 ± 0.04
K_M /V_{max} (s)	47.9 ± 5.2	61.8 ± 6.2	65.3 ± 6.9	72.9 ± 7.1	89.4 ± 11.8

Buffer: 100 mM sodium phosphate (pH 8.0) at 37 °C, [CT]$_0$ = 40 nM, [sucAAPFpNA]$_0$ = 0 – 768 μ M and macromonomer]$_0$ = 0 – 400 μM, Monitor Abs at 412 nm.

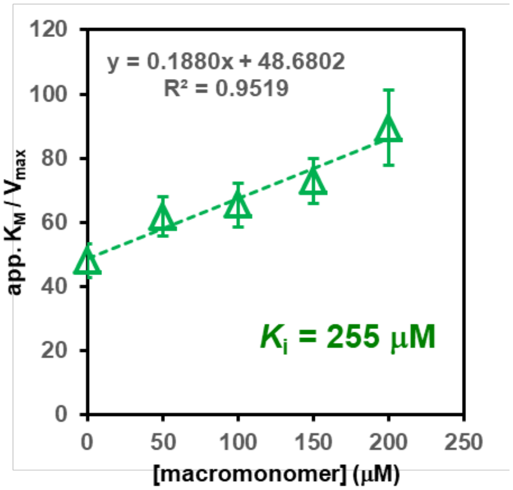

Figure S18. Michaelis-Menten Plots adding a macromonomer containing a TR cleavable and CT inhibiting peptides (IBAAPFSarPEG$_4$GPRGGOEGMA) to a native chymotrypsin solution.

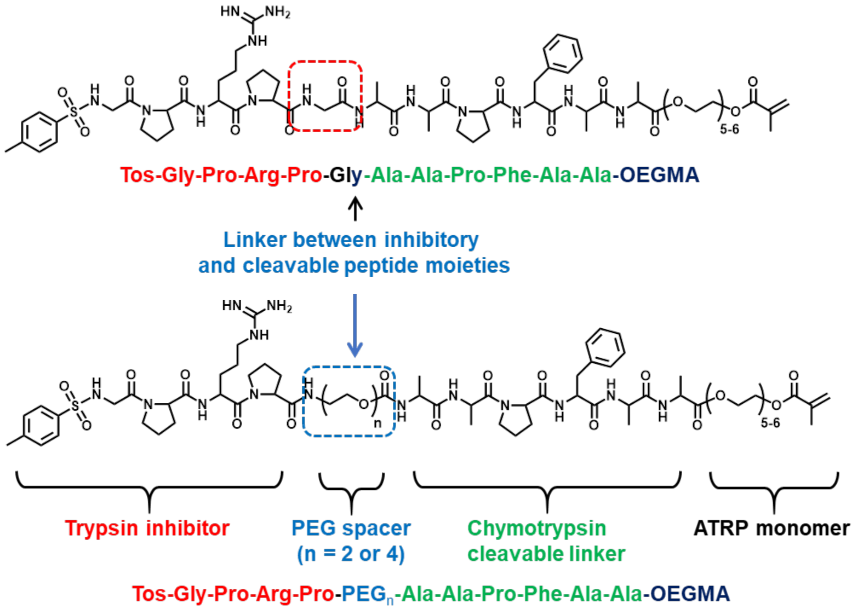

Figure S19. Chemical structure of macromonomers containing different linkers between the protease inhibitor and the cleavable peptide moieties.

Table S5. Characterization and enzymatic activity of TR-pCBMA and inhibited pro-TR polymer hybrids with different linkers between the inhibitory and cleavable peptide moieties.

	SEC-MALLS analysis				Michaelis-Menten parameters [d]		
samples	M_n [b] kg mol^{-1}	M_w [b] kg mol^{-1}	$Đ$ [b]	DP [c] (CBMA/inhibitor)	K_M (μM)	k_{cat} (s^{-1})	k_{cat}/K_M (μM^{-1} s^{-1})
TR-pCBMA	272.8	492.0	1.80	99.2 / 0	18.8 ± 2.8	45.7 ± 1.1	2.43 ± 0.36
TR-inhibitor (Gly)[a]	157.5	238.7	1.52	48.6 / 0.7	13.1 ± 1.4	30.4 ± 0.6	2.32 ± 0.25
TR-inhibitor (PEG_2)[a]	155.6	234.3	1.51	43.4 / 1.4	14.6 ± 1.8	24.7 ± 0.6	1.69 ± 0.21
TR-inhibitor (PEG_4)[a]	246.2	338.9	1.38	60.4 / 2.6	26.6 ± 1.2	23.2 ± 0.3	0.87 ± 0.09

Polymerization conditions: $[M]_0:[I]_0:[CuBr_2]_0:[HMTETA]_0:[NaAsc]_0$ = 50:0.5:5.0:0.5:6.0 (mM) in PBS and 10 vol% DMSO. 4 °C, 1 h. [M]; [CBMA] only or [CBMA]:[Macromonomer-inhibitor] = 9:1 molar ratio. [a] macromonomers containing the linkers in brackets were used. [b] determined by SEC-MALLS. [c] determined by SEC-MALLS and ^{1}H NMR spectra. [d] conditions: $[TR]_0$ = 8.4 nM, $[Tos\text{-}GPR\text{-}pNA]_0$ = 0 - 302 μM, buffer 100 mM Tris-HCl and 20 mM $CaCl_2$ (pH 8.0) at 37 °C.

Calculation of the concentration of inhibitor peptides in the activation of pro-protease hybrids

For pro-TR:

11.8 mg/mL of pro-TR hybrid was used. This is including 0.6 mg of TR and 11.2 mg of grafted polymer. From the molar ratio of CBMA (229 g/mol, DP = 60.4 / 63.0 per polymer chain) and macromonomer (1810 g/mol, DP = 2.6 / 63.0 per polymer chain) contained in the polymer, the

average molecular weight of the monomer unit was estimated to be 294 g/mol. The concentration of total monomer units was approximately 38.1 μmol/mL, of which the inhibitor peptide concentration was calculated to be 1.6 mM (4.1 mol% of macromonomer unit). After incubation with the trigger CT, approximately 70 mol% (1 – 0.8/2.6),of the inhibitor peptide was released to the activation solution, and its concentration was calculated to be 1.1 mM.

For pro-CT:

8.4 mg/mL of pro-CT hybrid was used. This is including 0.6 mg of CT and 7.8 mg of grafted polymer. From the molar ratio of CBMA (229 g/mol, DP = 39.7 / 42.5 per polymer chain) and macromonomer (1690 g/mol, DP = 2.8 / 42.5 per polymer chain) contained in the polymer, the average molecular weight of the monomer unit was estimated to be 326 g/mol. The concentration of total monomer units was approximately 23.9 μmol/mL, of which the inhibitor peptide concentration was calculated to be 1.6 mM (6.6 mol% of macromonomer unit). After incubation with the trigger TR, approximately 75 mol% (1 – 0.7/2.8) of the inhibitor peptide was released to the activation solution, and its concentration was calculated to be 1.2 mM.

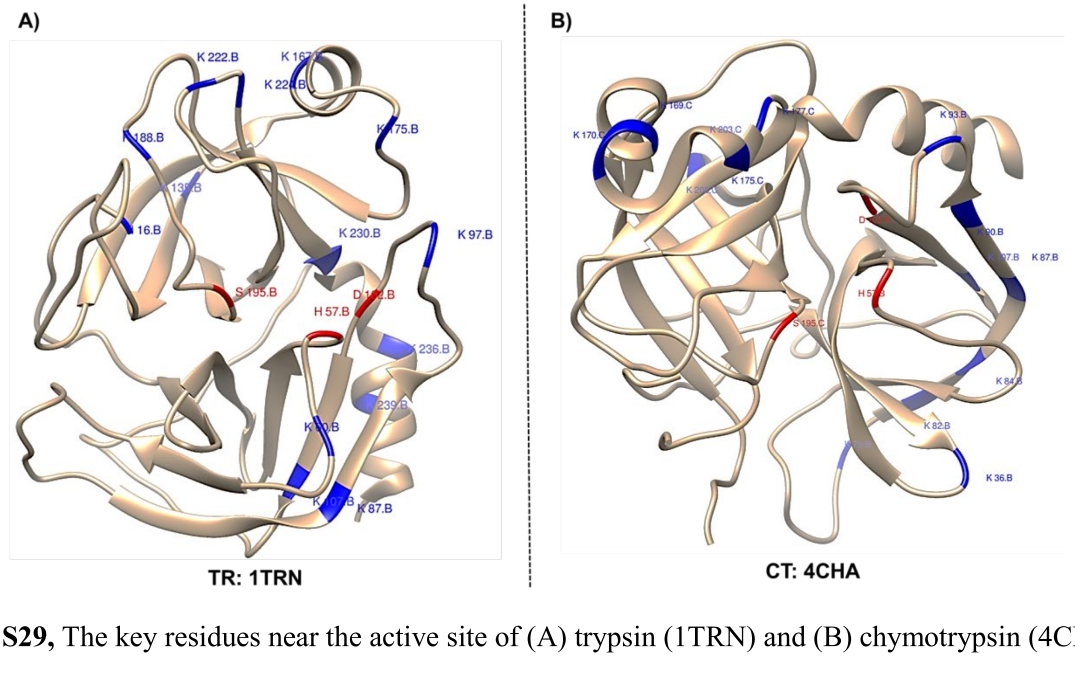

Figure S29, The key residues near the active site of (A) trypsin (1TRN) and (B) chymotrypsin (4CHA).

References

(1) Kaupbayeva, B.; Murata, H.; Rule, G. S.; Matyjaszewski, K.; Russell, A. J. Rational Control of Protein–Protein Interactions with Protein-ATRP-Generated Protease-Sensitive Polymer Cages. *Biomacromolecules* **2022**, *23*, 3831–3846.

(2) Murata, H.; Sanda, F.; Endo, T. Syntheses and radical polymerization behavior of methacrylamides having peptide moieties: Effect of the methylene chain introduced between the methacrylamide and peptide moieties on the polymerizability and polymer structure. *Macromolecules* **1997**, *30*, 2902-2906.

(3) Murata, H.; Sanda, F.; Endo, T. Synthesis and radical polymerization of a novel acrylamide having an α-helical peptide structure in the side chain. *Journal of Polymer Science Part a-Polymer Chemistry* **1998**, *36*, 1679-1682.
